# Beyond Poliomyelitis: A 21-Year Study of Non-Polio Enterovirus Genotyping and Its Relevance in Acute Flaccid Paralysis in São Paulo, Brazil

**DOI:** 10.3390/v16121875

**Published:** 2024-12-01

**Authors:** Rita Cássia Compagnoli Carmona, Fabricio Caldeira Reis, Audrey Cilli, Juliana Monti Maifrino Dias, Bráulio Caetano Machado, Daniele Rita de Morais, Adriana Vieira Jorge, Amanda Meireles Nunes Dias, Cleusa Aparecida de Sousa, Sabrina Bonetti Calou, Gabriel Henriques Ferreira, Lucas Leme, Maria do Carmo Sampaio Tavares Timenetsky, Maria Bernadete de Paula Eduardo

**Affiliations:** 1 Núcleo de Doenças Entéricas, Centro de Virologia, Instituto Adolfo Lutz, Secretaria de Estado da Saúde de São Paulo, Sao Paulo 01246-900, Brazil; 2 Divisão de Doenças de Transmissão Hídrica e Alimentar, Centro de Vigilância Epidemiológica “Prof. Alexandre Vranjac”, Secretaria de Estado da Saúde de São Paulo, Sao Paulo 01246-900, Brazil; 3 Centro de Virologia, Instituto Adolfo Lutz, Secretaria de Estado da Saúde de São Paulo, Sao Paulo 01246-900, Brazil

**Keywords:** enteroviruses, non-polio enteroviruses, acute flaccid paralysis, molecular epidemiology, pediatric patients

## Abstract

In the context of the near-global eradication of wild poliovirus, the significance of non-polio enteroviruses (NPEVs) in causing acute flaccid paralysis (AFP) and their impact on public health has gained increased attention. This research, conducted from 2001 to 2021, examined stool samples from 1597 children under 15 years in São Paulo, Brazil, through the AFP/Poliomyelitis Surveillance Program, detecting NPEVs in 6.9% of cases. Among the 100 NPEV-positive strains analyzed, 90 were genotyped through genomic sequencing of the partial VP1 region, revealing a predominance of *EV-B* species (58.9%), followed by *EV-A* (27.8%) and *EV-C* (13.3%). This study identified 31 unique NPEV types, including EV-A71, CVB2, and E11, as the most prevalent, along with the first documented occurrence of CVA19 in Brazil. These findings emphasize the importance of NPEV genotyping in distinguishing AFP from poliomyelitis, enhancing understanding of these viruses’ epidemiology. Moreover, it ensures that AFP cases are correctly classified, contributing to the effective surveillance and eradication efforts for poliomyelitis.

## 1. Introduction

Acute flaccid paralysis (AFP) is a clinical syndrome characterized by the sudden onset of weakness and paralysis in the proximal limb muscles, which is caused by polioviruses, including enteroviruses (EVs). In the post-polio era, non-polio enteroviruses (NPEVs) associated with AFP have become more prominent [1,2]. This diverse group includes coxsackieviruses (CVs), echoviruses (Es), enteroviruses (EVs), and rhinoviruses (RVs). In addition to their involvement in AFP, polioviruses exhibit a capacity to incite a diverse array of human pathologies on a global scale. These encompass a spectrum of conditions such as acute flaccid myelitis (AFM), myelitis, aseptic meningitis, encephalitis, myocarditis, pancreatitis, hand-foot-and-mouth disease (HFMD), severe acute respiratory syndrome, hemorrhagic conjunctivitis, severe neonatal sepsis, and persistent disorders such as type 1 diabetes [3,4,5]. 

Enterovirus constitutes a large genus within the *Picornaviridae* family, order *Picornavirales*, comprising over 100 distinct types. In humans, they are currently classified into four species: *Enterovirus alphacoxsackie* (formerly *Enterovirus A—EV-A*), *Enterovirus betacoxsackie* (*formerly Enterovirus B—EV-B*), *Enterovirus coxsackiepol* (*formerly Enterovirus C—EV-C*), and *Enterovirus deconjucti* (formerly *Enterovirus D—EV-D*). To ensure clarity of this article, we refer to the former nomenclature (*EV-A*, *EV-B*, *EV-C*, and *EV-D*). These classifications are based on phylogenetic relationships. *EV-A* species includes 25 types, such as CV and EV-A variants. EV-B encompasses 63 types, including Coxsackievirus B (CVB), Coxsackievirus A9 (CVA9), Echovirus (E), and EV-B virus. EV-C comprises 23 types, including polioviruses (PV1, PV2, and PV3), Coxsackievirus A (CVA), and EV-C variants. Finally, the *EV-D* species includes five types [6,7], contributing to the taxonomic and genetic diversity within this viral family. 

Genotyping NPEVs is important for epidemiological surveillance and outbreak investigation, as different serotypes may have different clinical presentations and transmission dynamics and may require different control measures [2]. By comparing the genetic sequences of enteroviruses isolated from different patients, we can determine whether the cases are linked to a common source of infection and identify the specific types of EVs involved. Studying enteroviruses can help identify emerging strains and subtypes, monitor their spread and evolution, and inform public health interventions, such as vaccine development and outbreak response [8]. 

Currently, the most common suggested cause of polio-like paralysis is NPEVs, and the main types are particularly Enterovirus D68 (EV-D68) and Enterovirus A71 (EV-A71) [2,9,10,11]. However, nearly every NPEV type has been identified from AFP patients, but the strength of association is much stronger with some types [1]. 

Nowadays, AFP is a general term for acute flaccid paralysis of the extremities and is a concept proposed to prove that polio has been eradicated in the region due to undetected poliovirus in stool samples. AFM has been proposed to avoid confusion with AFP. AFM is defined by the presence of AFP and a spinal cord lesion on magnetic resonance imaging that is primarily limited to the gray matter [10,12,13]. 

The circulation of NPEVs associated with AFP can vary by region and season. For example, the EV-A71 type belonging to the *EV-A* species has been implicated in several AFP outbreaks across Asia and Australia, while most types belonging to the *EV-B* species have been described in recent AFP surveillance reports from China, Spain, and West Africa [14,15,16]. Recently, EV-D68 has been associated with cases of AFP/AFM in children. This association was first described during an outbreak of AFM in the United States in 2014 [17,18]. Since then, sporadic cases have been reported worldwide, and accumulating evidence has strongly supported the association between EV-D68 and AFM [19,20,21,22,23,24,25]. 

In Brazil, the last case of wild poliovirus was reported in 1989. Following the last isolation of wild poliovirus in 1991 in Peru, the entire Americas Region was certified as polio-free in 1994, marking 30 years since this historic achievement (https://www.paho.org/en/campaigns/world-polio-day-2024, accessed on 24 October 2024). Although poliovirus has been eliminated in Brazil, AFP still occurs each year, which is not related to poliovirus. With wild poliovirus nearing eradication, NPEVs are likely filling the ecological niche left behind, a trend that may intensify with the cessation of bivalent oral polio vaccine (bOPV), increasing their role in AFP cases. [2]. In recent years, Brazil has faced challenges in maintaining high vaccination coverage due to factors such as vaccine hesitancy, inadequate access to health services in some regions, and misinformation about vaccine safety [26]. Despite these challenges, Brazil has continued to prioritize polio vaccination, and the country has not reported any cases of wild poliovirus since 1990. However, the occurrence of vaccine-derived poliovirus type 2 (VDPV2) in countries across the African continent and Southeast Asia (Indonesia) underscores the critical need for continued surveillance in post-eradication scenarios. The genotyping of NPEVs in the context of AFP surveillance holds profound significance at the world level. In the Brazilian context, the literature is notably sparse regarding instances of NPEV infections linked to AFP, and an exploration of their genetic diversity has been relatively limited [27,28,29]. 

Considering these knowledge gaps, the primary objective of this study was to characterize the types of NPEVs observed in individuals presenting with clinical symptoms resembling polio. This characterization is derived from a comprehensive 21-year surveillance effort conducted in São Paulo, Brazil. Additionally, this study seeks to explore the epidemiological foundations underlying AFP cases within the pediatric population. In parallel, it aims to highlight key findings from this investigation, which hold the potential to contribute significantly to the next phases of the ongoing poliomyelitis eradication campaign.

## 2. Materials and Methods

### 2.1. Selection of Archived Samples

This study was conducted between January 2001 and December 2021 in São Paulo State, south-eastern region of Brazil. At least one stool sample collected from 1597 children under 15 years of age from the AFP Surveillance Program was sent to the Enteric Diseases Laboratory, Virology Center, Adolfo Lutz Institute—State Reference Laboratory for Enteroviruses. Sample aliquots were stored at −20 °C. Subsequently, original vials of these specimens were sent to the National Reference Laboratory of AFP/Poliomyelitis, FIOCRUZ, Rio de Janeiro, to carry out the poliovirus research by cell culture isolation, as per the WHO algorithm [30]. Among 1597 cases of AFP, NPEVs were detected in 6.9% of the samples analyzed over the 21-year surveillance period. A total of one hundred (90.1%, n = 100/111) NPEV samples were selected for this study and further typed by the Enteric Diseases Laboratory, Adolfo Lutz Institute, using sequencing of the partial region of VP1 (viral protein 1). The criterion used for sample selection was the availability of specimens and enough amount for analysis.

### 2.2. Sample Processing and Virus Isolation

All stool samples NPEVs-positive stored −20 °C were prepared with 10% suspension in phosphate-buffered saline (PBS) with the addition of antibiotic solution (penicillin and streptomycin), and 200 μL were inoculated in two types of cell lines for isolation of viruses as follows: RD (human rhabdomyosarcoma, CCIAL-039) and HEp-2 (cells derived via HeLa contamination, contain HeLa marker chromosomes, ATCC-CCL-23). The cell lines were observed daily for the presence of viral cytopathic effect (CPE). When CPE was obtained, the infected cells were harvested and kept frozen at −70 °C.

### 2.3. NPEVs Typing by Reverse Transcription Polymerase Chain Reaction and Semi-Nested PCR

EV-positive isolates in cell cultures were collected and stored at −70 °C for RNA extraction. EV single-stranded RNA was extracted from 140 μL of cell culture supernatant using the QIAamp Viral RNA Mini kit (Qiagen, Valencia, CA, USA), according to the manufacturer’s instructions. All the isolates were subjected to reverse transcription polymerase chain reaction (RT-PCR) to amplify the partial VP1 viral capsid gene region [31,32]. Briefly, amplification was performed in a 500 μL RT-PCR reaction containing deoxynucleotides (1.25 mM each), MgCl_2_ (1.25 mM), dithiothreitol (0.1 M), 5× buffer (Tris-HCl 300 mM), 10× buffer (Tris-HCl 100 mM), and 20 μM primers 292–222 (338 bp product); 1U SuperScript III-Reverse Transcriptase (Invitrogen, Waltham, MA, USA); 2 U Platinum Taq DNA Polymerase (Invitrogen); and RNAsin (Invitrogen). The RT-PCR and snPCR products were analyzed using 1.5% agarose gel electrophoresis and stained with GelRed Nucleic Acid Gel Stain (Biotium, Inc., Fremont, CA, USA). NPEV stool sample negatives in cell culture were subjected to RNA extraction using the QIAamp Viral RNA Mini kit (Qiagen, Valencia, CA, USA), cDNA synthesis, and a semi-nested PCR assay using primers AN88 and AN89 [32].

### 2.4. Partial Sequencing of the VP1 Region

DNA amplicon products were purified using a PureLink PCR Purification Kit (Invitrogen) and submitted to sequencing reactions using Big Dye Terminator Ready Reaction Mix (version 3.1, Applied Biosystems) with the same primer set used in the RT-PCR and RT-snPCR reactions. Dye-labeled products were sequenced using an ABI 3500 sequencer (Applied Biosystems, Foster City, CA, USA). Sequencing chromatograms were edited manually using Sequencher™ 4.1.4 software (Gene Codes Corporation, Ann Arbor, MI, USA), and the genotype was determined by using the web-based open access typing tools for enteroviruses, Enterovirus Genotyping Tool Version 2.6.1, available at https://www.genomedetective.com/app/typingtool/etv/ [33]. 

### 2.5. Phylogenetic Analysis

We performed alignment of the sequences using the BioEdit software (v. 7.2.5.) [34] (Hall 1999). The best-fit nucleotide substitution model (GTR+Γ+I) was carried out by the Akaike information criterion available in jMODELTEST (v.2.1.10). The evolution rates were determined based on partial VP1 gene sequence (*EV-A*: 315 bp, *EV-B*: 335 bp, and *EV-C*: 340 bp) data by the Bayesian Markov Chain Monte Carlo (Bayesian MCMC) method implemented in BEAST (v1.10.4) [35] with 1 × 10^7^ states and sampled every 1 × 10^4^ states. The generated probabilities were analyzed using Tracer (v. 1.5) [36]. The final maximum clade credibility tree was generated using BEAST’s TreeAnnotator with a burn-in of 2 × 10^7^. We visualized phylogenetic trees with the ggtree package [37] (Yu et al. 2017) in R (v4.3.1) [38].

### 2.6. Nucleotide Sequence Accession Numbers

Nucleotide sequences determined in this study were submitted to GenBank under the accession numbers OK605910 to OK605916, OL771248 to OL771253, OR454480, OR498905, OR504975 to OR504997, and OR508976 to OR508985.

### 2.7. Ethical Statement

This study was carried out in accordance with the guidelines of the Declaration of Helsinki and received approval from the Ethics Committee of the Adolfo Lutz Institute under Approval Number 1.719.525, dated 9 September 2016.

## 3. Results

### 3.1. NPEVs Detection

Stool samples were obtained from 890 (55.7%) male 704 (44.1%) female patients with AFP and 3 newborns, whose gender was not indicated. In total, 111 (6.9%) samples were positive for NPEVs by isolation in RD or Hep-2C cell culture but remained negative in L20B cells, performed by the National Reference Laboratory of AFP/Poliomyelitis, FIOCRUZ, Rio de Janeiro, Brazil (Table 1). According to the criterion used for sample selection in this study, it was possible to select 100 NPEVs (90.1%) for carrying out genotyping between the years 2001 and 2021 (Table 1). 

**Figure 1:**
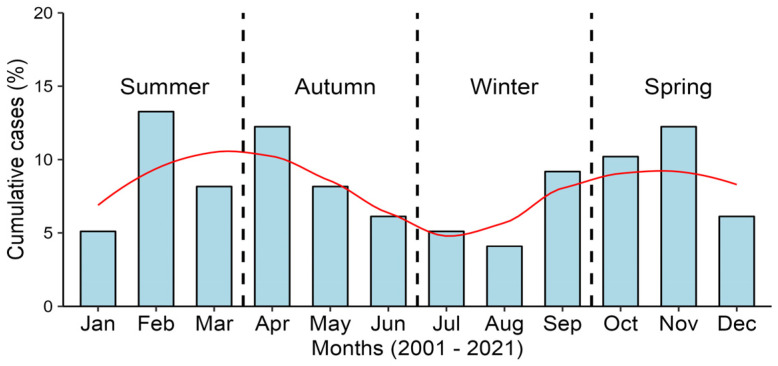
Cumulative cases (%) of NPEV-positive cases in the AFP/Poliomyelitis Surveillance Program by month during 2001–2021.

NPEV-positive cases of AFP were identified consistently over the years, with detection rates ranging from 2.6% (1/38) in 2021 to 12.5% (8/64) in 2013. Notably, no NPEV detections occurred in 2017, 2018, and 2020. While the absence of cases in 2020 coincides with the onset of the COVID-19 pandemic, the earlier gaps in detection (2017 and 2018) are unrelated to the pandemic and may reflect other epidemiological or methodological factors. Seasonal trends indicate two distinct peaks in detection rates, with higher occurrences observed during the summer months, particularly in February, and during the spring months, especially in November. A smaller increase is also observed in April, during the autumn season. These seasonal patterns underscore the importance of maintaining consistent surveillance efforts throughout the year to monitor NPEV circulation effectively, as illustrated in Figure 1.

The age distribution of patients who tested positive for NPEVs ranged from four months to 13 years, culminating in a median age of 40.2 months (equivalent to 3.4 years old), as presented in Table 2.

### 3.2. NPEVs Typing 

**Figure 2:**
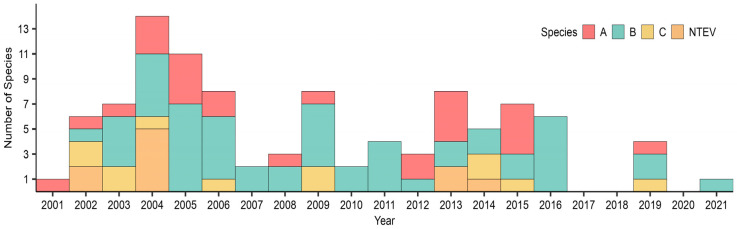
Distribution of *EV-A*, *EV-B,* and *EV-C* species, from 2001 to 2021. NTEV: not type EV.

Out of 100 NPEVs-positive cases analyzed, 90 (90.0%) could be genotyped. Of those, 53 (58.9%) were assigned to *EV-B* species, 25 (27.8%) were assigned to *EV-A*, and 12 (13.3%) were assigned to *EV-C*, which comprised 17, 9, and 5 genotypes, respectively (Figure 2). Ten samples were not typed EV (NTEV), and no *EV-D* were detected. 

Throughout the 21 years of surveillance, *EV-B* emerged as the prevailing species. However, there were specific instances when the dominance shifted to the *EV-A* species, accounting for the sole type identified in 2001 (66.7%, n = 04/06) and contributing to 57.1% (04/07) of cases in 2013 and 2015, respectively. Additionally, the year 2002 witnessed the *EV-C* species spearheading the scenario, responsible for 50.0% (2/04) of NPEVs detections (Figure 2). 

Between serotypes of *EV-B* species, E11 and CVB2 were the most prevalent (17.0% each, n = 09/53), followed by E6 (13.2%, n = 07/53), CVB3 (11.3%, n = 06/53), CVB5, and E30 (7.5% each, n = 04/53). EV-A71 was the most prevalent of *EV-A* (36.0%, n = 09/25), followed by CVA16 (20.0%, n = 05/25) and CVA4 (16.0%, n = 04/25). Among the *EV-C* types, EV-C99 emerged as the predominant subtype, constituting 50.0% (n = 06/12) of the cases, followed by CVA19 at 25.0% (n = 03/12) (Table 3).

This study documented the presence of 31 distinct NPEV types throughout the analyzed period. Specifically, EV-A71, CVB2, and E11 held prominence as the primary circulating NPEVs. Moreover, this study, since the beginning of NPEV surveillance, provided the first evidence of CVA19 in Brazil (Table 3).

*EV-A* species exhibited a higher prevalence among younger patients, with a median age of 27.7 months, compared to *EV-B* (median age of 42 months) or *EV-C* (median age of 38 months). The youngest median age was noted among patients affected by E18 (6 months) and CVA2 (9 months).

### 3.3. Phylogenetic Analysis of NPEVs

**Figure 3:**
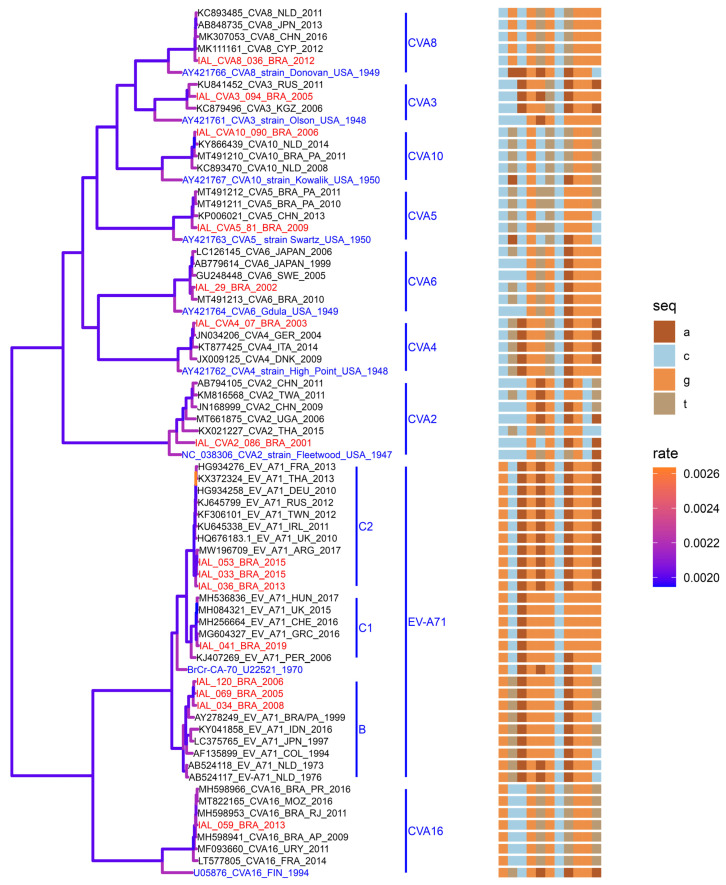
Multiple-sequence-alignment analysis (a, c, g, and t nucleotide window: 150–175) and maximum clade credibility (MCC) tree from partial sequences (VP1) of *EV-A* species: EV-A71 (321 bp), CVA2 (304 bp), CVA3 (301 bp), CVA4 (306 bp), CVA5 (300 bp), CVA6 (294 bp), CVA8 (329 bp), CVA10 (332 bp), and CVA16 (335 bp). The genotype was defined as more than 75% nucleotide similarities in the VP1 region. GenBank reference strains are highlighted in blue. Genotypes isolated in this study are highlighted in red. The rate of nucleotide substitution (Bayesian MCMC) is shown by the color gradient in the phylogenetic tree.

**Figure 4:**
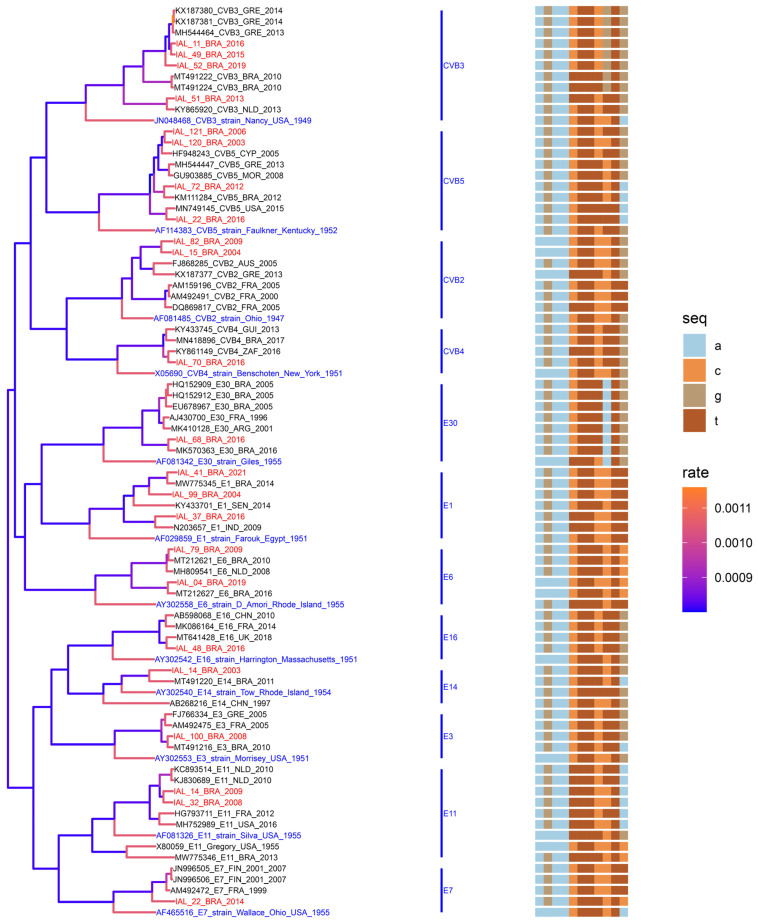
Multiple-sequence-alignment analysis (a, c, g, and t nucleotide window: 150–175) and maximum clade credibility (MCC) tree from partial sequences (VP1) of *EV-B* species: CVB2 (331 bp), CVB3 (327 bp), CVB4 (322 bp), CVB5 (33 bp), E1 (334 bp), E3 (319 bp), E6 (332 bp), E7 (334 bp), E9 (309 bp), E11 (33 bp), E13 (334 bp), E14 (317 bp), E16 (317 bp), E18 (311 bp), E25 (334 bp), E30 (318 bp). The genotype was defined as more than 75% nucleotide similarities in the VP1 region. GenBank reference strains are highlighted in blue. Genotypes isolated in this study are highlighted in red. The rate of nucleotide substitution (Bayesian MCMC) is shown by the color gradient in the phylogenetic tree.

**Figure 5:**
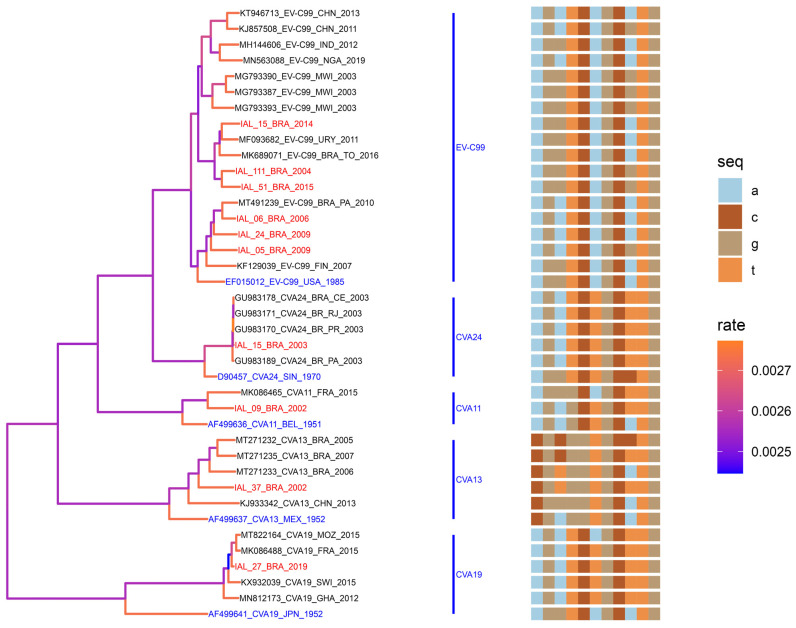
Multiple-sequence-alignment analysis (a, c, g, and t nucleotide window: 150–175) and maximum clade credibility (MCC) tree from partial sequences (VP1) of *EV-C* species: EV-C99 (333 bp), CVA11 (363 bp), CVA13 (338 bp), CVA19 (332 bp), and CVA24 (364 bp). The genotype was defined as more than 75% nucleotide similarities in the VP1 region. GenBank reference strains are highlighted in blue. Genotypes isolated in this study are highlighted in red. The rate of nucleotide substitution (Bayesian MCMC) is shown by the color gradient in the phylogenetic tree.

Based on reliable partial VP1 genomic sequencing (~300 bp), phylogenetic analysis was performed on 48 NPEVs, which comprised 15 *EV-A*, 23 *EV-B*, and 10 *EV-C* types. The newly generated nucleotide sequences were subsequently aligned with 145 sequences sourced from GenBank (available at https://www.ncbi.nlm.nih.gov/), detailed in Supplementary Table S1. Phylogenetic trees, illustrating the evolutionary relationships among *EV-A*, *EV-B*, and EV-C species, are presented in Figure 3, Figure 4 and Figure 5, respectively. 

#### 3.3.1. Phylogenetic Analysis of NPEVs Within the EV-A Species

Within the *EV-A* species, the EV-A71 type emerged as the most prevalent. These findings include recent sequences sourced from Sao Paulo, Brazil, which were assigned to subgenogroup C1 (represented by IAL-041/2019) clustered alongside human isolates from Greece (MG604327/2016), Hungary (MH536836/2017), Peru (KJ407269/2006), Switzerland (MH256664/2016), and the United Kingdom (MH084321). This grouping exhibited a high similarity at the nucleotide level (nt) of 92% to 98% (100% aa). Similarly, another cluster was identified as subgenogroup C2, encompassing IAL-36/2013, IAL-33/2015, and IAL-53/2015. These viruses clustered with isolates from Argentina (MW196707) detected in wastewater samples in 2017 and with isolates from France (HG934276/2013), Germany (HG934258/2010), Ireland (KU645338/2011), Russia (KJ645799/2012), Thailand (KX372324/2013), and Taiwan (KF306101/2012) detected in human samples, displaying a high nt similarity range of 94% to 98% (99–100% aa). In contrast, isolates obtained in earlier years were classified within genogroup B (IAL-69/2005, IAL-120/2006, and IAL-034/2008), sharing a clustering pattern with isolates from Brazil (AY278249/1999), Colombia (AF135899/1994), Indonesia (KY041858/2016), Japan (LC375765/1997), and The Netherlands (AB524117/1976 and AB524118/1973), demonstrating a nt similarity of 88% to 92% (99% to 100% aa) (Figure 3). Genogroups B and C (subgenogroups C1 and C2) included isolates representative of different continents, suggesting a global distribution (Figure 3). 

The remaining sequences within the *EV-A* species (CVA2 to CVA6, CVA8, CVA10, and CVA16) exhibited distinct clustering patterns, encompassing representatives from diverse geographical origins. The Brazilian CVA2 (IAL-086-BRA/2001) isolate displayed low aa genetic homology to other CVA2 strains detected in Asia (AB794105, KM816568, and KX021227) and Africa (MT661875) (50.2% aa), but in the phylogenetic tree, the Brazilian strain clustered with the reported strains (Figure 3). 

The CVA4 IAL-07-BRA/2003 isolate had high homology among strains from Europe (JX009125/2009, Denmark; KT877425/2014, Italy; and JN034206/2004, Finland). This is the first report of genome sequencing of CVA4 in Brazil (Figure 3). 

To date, the GenBank database has not featured CVA3 and CVA8 nucleotide sequences originating from South America, and this is the first report. CVA3 (IAL-094-BRA/2005) constituted a cluster composed of strains from Kyrgyzstan (KC879496/2006) and Russia (KU841452/2011), sharing a high sequence nt similarity of 88.0% and 90.0% (96.0% and 97% aa), respectively. CVA8 (IAL-036-BRA/2012) exhibited a unique cluster encompassing strains originating from Asia (MK307053/2016, China; MK111161/2012, Cyprus; and AB848735/2013, Japan) and Europe (KC893485/2011, The Netherlands) with a nt 98.8% similarity and 96.5% aa (Figure 3). 

#### 3.3.2. Phylogenetic Analysis of NPEVs Within the EV-B Species

The phylogenetic analysis of NPEVs within the *EV-B* species focused on the most prevalent types identified in this study: CVB2, E11, E6, CVB5, and E30. The analysis incorporated temporal information by including strains from different years and geographic regions. 

For CVB2, the analysis confirmed a close relationship between the strains identified in this study and European isolates from France (2000 and 2005), Austria (2005), and Greece (2013). Brazilian CVB2 sequences formed a distinct clade yet remained closely intertwined with European strains, suggesting the emergence of a distinct lineage (Figure 4). The E11 isolates exhibited nucleotide similarities ranging from 79.0% to 94.0% (mean 97.0% amino acid identity) when compared to strains from France (2002), The Netherlands (2010), and the United States (2016). However, a comparison with a Brazilian strain revealed lower nucleotide similarity (70.0%) but relatively high amino acid identity (90.0%) (Figure 4). For E6, the isolate IAL-79-BRA/2009 showed greater nucleotide similarity with strains from Brazil (2010) and The Netherlands (2009) compared to a more recent Brazilian strain (MT212627_E6_BRA_2016, 70.0% nucleotide similarity). In contrast, the IAL-04-BRA/2019 isolates exhibited an inverse pattern, highlighting genetic diversity within E6 and varying genetic relatedness across different years (Figure 4). CVB5 isolates exhibited substantial genetic identity (85–95% nucleotide similarity and >99.0% amino acid similarity) with strains from Brazil (2012), Cyprus (2005), Greece (2013), Morocco (2008), and the United States (2015). These findings underscore the genetic relatedness of CVB5 from this study with strains reported globally between 2005 and 2015 (Figure 4). For E30, the analysis of IAL-68-BRA/2016 revealed a distinct clade formed exclusively with another Brazilian strain from the same year (MK570363_E30_BRA_2016). Comparisons with older strains from Argentina (2001), Brazil (2005), and France (1996) showed amino acid similarity ranging from 61.0% to 67.0% (Figure 4). The remaining EV-B species sequences, including CVB3, CVB4, E1, E3, E7, E14, and E16, formed distinct clusters representing diverse geographic origins, with nucleotide similarities ranging from 80% to 98% (Figure 4). This study provides the first report of genome sequencing of E16 in South America.

#### 3.3.3. Phylogenetic Analysis of NPEVs Within the EV-C Species

EV-C99 and CVA19 were the most prevalent within the *EV-C* species. The partial sequences of EV-C99 that we have elucidated demonstrate a nt similarity ranging from 80% to 85% when compared to the prototype strain (USA-Ok85-10627, 94.6% aa). Additionally, upon conducting a comparative assessment with representative EV-C99 strains identified in Malawi (2003), Finland (2007), Uruguay (2011), Nigeria (2019), China (2011 and 2013), and Brazil (2016 and 2019), we observe nt similarities ranging from 79.2% to 84.9% (91.6% to 94.8% aa) (Figure 5). 

Genotyping EV described in the present investigation has provided the ability to identify the CVA19 for the first time in Brazil. The phylogenetic analysis of the IAL-27-BRA/2019 strain had shown nt similarity with representative CVA19 strains (90% to 96% and 72% to 86% aa) identified in Ghana (2012), Mozambique (2015), France (2015), and Switzerland (2015) and a nt similarity of 71% when compared to the Japan prototype strain (8663 Dohi-1952, 34.0% aa) (Figure 5). 

CVA11 demonstrated a nt similarity of 82% (53.0% aa) with the French sample (2015). Phylogenetic analysis of the CVA13 isolate, IAL-37-BRA/2002, situated it within a cluster of Brazilian strains, demonstrating an 80.3% nucleotide similarity, despite the low genetic homology (aa 41.0% to 49.0%), and it showed a 78.0% nt similarity to a sample from China (aa 45%). In contrast, CVA24 demonstrated a remarkable sequence nt similarity exclusively with representative Brazilian strains, ranging from 97% to 98% (99.0% aa) (Figure 5).

### 3.4. Epidemiological Data

The final diagnosis among patients with AFP was diverse; Guillain-Barré syndrome (GBS) was the most prevalent, identified in 29 cases (29.0%), followed by encephalitis/meningoencephalitis in 15 cases (15.0%). Unspecified paralytic syndrome was noted in 11 cases (11.0%), other undefined nervous system disorders in 5 cases (5.0%), unspecified polyneuropathy in 5 cases (5.0%), and periodic paralysis in 4 cases (4.0%). An assortment of other clinical syndromes, including acute flaccid myelitis, malignant neoplasm of the central nervous system (CNS), myelopathy, neuroviral, stroke, unspecified intoxication, and myoneural disorder, collectively accounted for the remaining 11 cases (11.0%). Twenty cases did not have clinical diagnosis information.

Information regarding the progression of paralytic disease (after 60 days from symptom onset) was obtained from 72 patients among 100 NPEVs-positive cases in this study. Twenty-two patients had sequelae after 60 days from the onset of symptoms (22.0%). Fifteen distinct genotypes (CVA2, CVA5, CVA6, CVA24, and CVB2 to CVB5, E7, E9, E11, E14, E30, EV-A71, and EV-C99) were associated with severe cases and led to sequelae. Supplementary Table S2 provides a summary of the EV types identified in relation to disease evolution.

## 4. Discussion

The global incidence of poliomyelitis has significantly decreased over the past three decades, with numerous countries successfully interrupting wild poliovirus transmission. Nonetheless, persistent cases are reported in regions such as Pakistan and Afghanistan. As the global eradication of wild poliovirus approaches, NPEVs are likely filling the ecological niche it leaves behind, a shift that may intensify with the cessation of bOPV, increasing their relevance in AFP as well as in other diseases of public health importance [2,4].

Our 21-year study provides significant findings on the prevalence and diversity of NPEV genotypes detected in children under 15 years of age hospitalized with AFP in the state of São Paulo, Brazil. NPEVs isolation rate from AFP cases was 6.9% of the samples analyzed, lower than 10% suggested by WHO to the reference laboratories [28]. However, this prevalence varied annually, with rates as low as 2.6% in 2021 and reaching 12.5% in 2013. These results show that the average NPEVs detection rate is similar to that reported in two surveillance studies in Brazil [28,29] and is comparable to findings in other countries, such as Iran, Italy, and Tunisia [39,40,41]. On the other hand, the average frequency of NPEVs isolation in countries such as Ghana (20.0%), Somalia (19.2%), and India (19.5%) exhibited substantially higher rates compared to the samples analyzed in our current study [42,43]. The absence of NPEV detection during the COVID-19 pandemic is consistent with reports from multiple countries observing a significant reduction in enterovirus circulation due to public health measures like lockdowns and social distancing [23,44,45]. 

In this study, seasonal trends revealed two distinct peaks in detection rates, with higher occurrences observed during the summer and spring seasons. This contrasts with countries in temperate climates, where EV infections typically show a single peak, predominantly during the summer [46]. These seasonal patterns highlight the importance of maintaining consistent surveillance efforts throughout the year to effectively monitor NPEV circulation. 

In Brazil, there is a limited number of studies elucidating the molecular diversity and circulation patterns of NPEVs in cases of AFP [27,28,29]. This scarcity underscores the importance of continued research and surveillance to ascertain the epidemiological landscape of NPEVs in the region.

Our findings indicate a prominent susceptibility to NPEV infections among the pediatric population aged under 5 years. Children aged between 2 and 5 years accounted for the highest percentage of NPEVs cases at 42.3%. This is likely due to their increased exposure to external environments, like starting school, which coincides with heightened social interactions and potential virus transmission [47]. The prevalence of NPEVs infection reduces considerably in the age bracket of 6 to 15 years, contributing to only 18.0% of the total cases. This reduced susceptibility could be attributed to acquired immunity from previous infections or exposures [48].

The genotyping of NPEVs throughout two decades in São Paulo State, Brazil, yielded valuable information regarding the distribution and epidemiological patterns of these viruses. The predominance of *EV-B* species, accounting for 58.9% of all genotyped cases, aligns with global trends [15]. 

EV-A71, CVB2, and E11 were the most common serotypes identified among patients with AFP in our study. Additionally, the emerging types EV-C99, CVA16, and CVA19 were detected. Considering the diversity of genotypes identified in this study, we will discuss the most frequently detected types, as well as those considered emergent within our setting.

The predominance of *EV-B* species among NPEV-positive cases is consistent with global trends, where this species is frequently identified in AFP surveillance. This underscores the need for continuous monitoring of *EV-B*, which remains one of the most commonly detected enterovirus species in many regions [48,49].

The identification of 31 distinct NPEVs, including the first detection of CVA19 in Brazil, enhances our understanding of enterovirus diversity in the region. Emerging strains reflect ongoing evolution and adaptation, as observed in various global settings [50,51].

The NPEV genotypes detected in our study in cases of AFP have also been associated with various other diseases. These include CNS disorders (CVA2 to CVA6, CVA8, CVA10, CVA11, CVA13, CVA16, CVA19, CVA24, CVB1 to B5, E1, E3, E6, E9, E11, E13, E14, E16, E18, E25, E30, and EV-A71). Additionally, they have been associated with myocarditis/pericarditis (CVA16, CVB1 to B5, E1, E3, E6, E7, E9, E11, E13, E16, and EV-A71), pleurodynia (CVA2, CVA4, CVA6, CVA10, CVA16, CVB1 to B5, E1, E3, E6, E7, E9, E11, E14, E16, E18, E25, and E30), and respiratory tract infections (CVA16, CVA24, CVB1 to B5, E1, E3, E6, E7, E9, E11, E13, E14, E16, E18, E15, E30, and EV-A71). Furthermore, these genotypes have been implicated in HFMD (CVA4 to CVA6, CVA10, CVA16, CVB1 to B5, E9, E11, and EV-A71), herpangina (CVA2 to CVA6, CVA8, CVA10, CVA16, CVA19, CVB1 to CVB6, E3, E6, E9, E11, E16, E25, and EV-A71), and hemorrhagic conjunctivitis (E7 and CVA24). They are also associated with gastroenteritis (CVA19, CVB2 to CVB5, E1, E6, E7, E11, E12, E14, E18, E25, E30, EV-A71, and EV-C99), hepatitis (CVB1 to CVB5, E3, E6, E7, E9, E11, E14, E25, and E30), pancreatitis (CVA24, CVB1 to CVB5, E6, E11, and EV-A71), and type 1 diabetes (CVB1 to CVB5, E3, E6, E9, E11, E16, E18, E25, E30, and EV-A71) [5,52].

EV-A71, a member of the *EV-A* species identified in samples from our study, displayed phylogenetic clustering within genogroups B (isolates from 2005, 2006, and 2008), C1 (2019), and C2 (2013 and 2015) through genetic analysis. This is consistent with studies documenting the global spread of EV-A71, particularly the C1 and C2 subgenogroups [53,54,55]. Currently, EV-A71 is categorized into four genogroups, designated as A, B, C, and D. Genogroups B and C are further subdivided into subgenogroups B1-B5 and C1-C5, respectively [56]. Subgenogroups B1 and B2 were predominant in the Americas and Europe during the 1970s and 1980s, while C1 became predominant from the 1990s onward [53,57], followed by C2 between 2000 and 2010 in the Philippines [58]. Our data are partially consistent with another study conducted in Brazil, which documented the circulation of EV-A71 in approximately 8% of AFP cases associated with enteroviruses between 2005 and 2017. To date, only EV-A71 genogroup B has circulated in Brazil in 1999 and from 2009 to 2014, while the subgenogroup C2 was identified in AFP cases during 2015 and 2016 [29,59]. Therefore, our study marks the first detection of the EV-A71 C1 in Brazil. The first identification of a C1 in South America was reported in samples from Argentina in 2017 [60]. EV-A71 subgenogroup C2 has been circulating in other South American countries since 2011 in wastewater samples, such as Argentina and Uruguay [60]. The EV-A71 C genogroup was first detected in the late 1980s and has demonstrated more widespread circulation than the B genogroup, particularly across the United States, the Western Pacific region, and several European countries [53,55,57,61]. The geographic distribution of other subgenogroups within genogroup C appears to be restricted, as subgenogroups C3, C4, and C5 have been observed exclusively in Asian countries [58].

EV-A71 is frequently associated with HFMD, including severe forms of the illness, and is strongly linked to serious and sometimes fatal CNS infections. Consequently, EV-A71 is currently regarded as an emerging enterovirus with significant implications for human health [60,62]. Ongoing efforts in molecular epidemiology and vaccine development are focused on its containment and management [54,63,64].

CVB2, a member of the *EV-B* species, is also associated with a wide range of human diseases, varying from mild, self-limiting conditions to severe acute and chronic disorders, and detected in AFP [65]. CVB2 is also a significant cause of aseptic meningitis, especially in neonates and infants. Although rare, cases of encephalitis and meningoencephalitis have also been reported [66]. CVB2 was identified in a fatal case of meningoencephalitis in Brazil, involving a healthy adolescent. This case highlighted the genetic relationship between the isolated CVB2 strain and other global isolates [67]. Outbreaks of CVB2 are infrequent, and much of the existing research is derived from case reports, although an outbreak occurred in Israel in 2022, where 14 children suffering from meningoencephalitis tested positive for CVB2. Two of these children presented with ataxia and imaging features of rhombencephalitis, a complication not previously described in association with CVB2. This suggests an evolving clinical profile for CVB2 infections, underscoring the need for heightened surveillance and further study into the neurological impacts of this virus [68]. CVB2 has been identified in 1.3% to 6.4% of surveillance data for documented enterovirus serotypes and consistently ranks among the 15 most commonly isolated enterovirus serotypes in the United States (https://www.cdc.gov/ness/data-vis/, accessed on 28 October 2024). Although CVs have been linked to various disorders, the true impact of these pathogens on human health, particularly on the CNS and the development of neurological diseases, requires further understanding [66,67].

E11, a member of the *EV-B* species, was also the most common genotype found in cases of AFP in this study. In global studies conducted on the surveillance of NPEVs in cases of AFP, E11 has also been identified following EV-A71 [1]. In Brazil, it has been detected in children under one year of age with AFP [29]. Echoviruses have recently been identified in Brazil in stool samples from patients with gastroenteritis [69,70]. In the phylogenetic analyses, E11 isolates clustered with strains from Europe, the United States, and Brazil, indicating the possible existence of distinct lineages or sublineages (see Supplementary Table S1 for strain references). Despite its common occurrence, there remains a substantial lack of genetic data for E11 in Brazil. This necessitates a comprehensive study of the molecular epidemiology of E11 across the country, particularly considering the extensive geographic regions. 

In the limited studies conducted in Brazil, *EV-B* types are generally associated with CNS diseases [66,71,72] and have also been reported in meningitis outbreaks, primarily caused by E6 (in 2004) and E30 (during 1998–1999 and 2013–2017) [71,73,74]. However, none of the AFP cases in this study were linked to some outbreaks. In our study, E6 and E30, along with CVB3, were the most detected types after CVB2 and E11, highlighting their potential role in AFP. EV-C99 isolates, belonging to the *EV-C* species, emerged in this study starting in 2004 and exhibited high genetic similarity with strains from countries across different continents, including Malawi, Finland, and China, emphasizing the global circulation and genetic diversity of enteroviruses. This aligns with studies that have documented the widespread distribution and significant genetic variability within the *EV-C* species [75,76].

EV-C99 strains have been isolated from cases of AFP [77,78], although the virus is known for its silent circulation, associated with asymptomatic or mild infections that often go [79]. However, in this study, EV-C99 was linked to cases of AFP, a severe manifestation of the disease, with one case presenting sequelae after evaluation 60 days from symptom onset. This EV type was first identified in South America in 2013, in a fecal sample from a child with acute diarrhea in the state of Tocantins, northern Brazil [79]. In cases of AFP, EV-C99 was first detected in Brazil in 2005, during a study conducted between 2005 and 2017 aimed at characterizing NPEVs, revealing a prevalence of 3.8% [29]. Subsequently, it was detected again in 2010–2011, 2018, and 2021 in fecal samples from Brazilian children, both presenting with diarrhea [71,80]. 

The first detection of CVA19 in this study and its phylogenetic relationship with strains from Africa and Europe highlight the interconnected nature of global enterovirus transmission (see Supplementary Table S1 for strain references). CVA19 is considered a rare EV and has been associated with neurological diseases, such as meningitis, as well as herpangina, gastroenteritis, and, more recently, HFMD [5,81]. It has frequently been isolated from human fecal samples, underscoring its prevalence in the human gastrointestinal tract [76].

In the analysis of sequelae cases among children with AFP, a diverse range of viral genotypes was identified. Genotypes with multiple occurrences included E11 (2 cases), CVB3 (2 cases), and CVB2 (2 cases), suggesting a strong association with AFP in these patients. Other genotypes observed in single cases included CVA2, E7, EV-A71 C2, CVB5, CVA5, EV-C99, E30, CVA6, E30-V, CVA24 IV, E14, and E9. The detection of EV-C99, along with genotypes such as E7, E9, E11, E12, and E30, underscores the genetic variability among enterovirus species associated with AFP cases with sequelae. Notably, EV-A71, which was also detected, has well-documented evidence of a causal role in AFP.

AFP has been linked to various NPEVs. However, a definitive causal association between genotype and disease severity has been well established only for EV-D68 and EV-A71. Other genotypes have been reported in cases of AFP and AFM, but their association with disease progression and severity is less common [14,19,82].

In this analysis, 16 out of the 22 children (about 73%) with sequelae were under five years of age. This concentration of cases in younger children suggests a heightened susceptibility to severe outcomes in this age group.

We, the authors, presume that multiple factors may have influenced disease progression, leading to sequelae in these children. Individual variations in immune response could play a critical role, with factors such as an underdeveloped or compromised immune system, genetic predispositions to severe outcomes from enterovirus infections, and limited access to timely treatment all potentially contributing to disease progression and an increased likelihood of sequelae, particularly in resource-limited settings.

## 5. Conclusions

Our study highlights the diversity and persistent circulation of NPEVs in AFP cases among children under 15 years in São Paulo, Brazil, over two decades. The predominance of *EV-B* species and identification of diverse genotypes, including the first report of CVA19 in the country, reflect the dynamic evolution of these viruses. The detection of EV-A71 and emerging viruses underscores the need for enhanced surveillance. Phylogenetic analyses revealed links between Brazilian and global strains, emphasizing the widespread dissemination of NPEVs.

In Brazil, the lack of systematic surveillance for NPEVs in AFP and related syndromes limits understanding of their circulation and public health impact. Implementing such a program could provide essential data to inform preventive strategies and improve public health responses.

## Figures and Tables

**Table 1:** Non-polio enteroviruses detected in suspected cases of AFP/poliomyelitis in the State of São Paulo, Brazil, and selected for molecular characterization into genotypes from 2001 to 2021.

Year	AFP Cases with Stool Samples	Positive Cases NPEVs		Selected NPEV Samples *	
	n=	n=	%	n=	%
2001	72	8	11.1	1	12.5
2002	85	6	7.1	6	100.0
2003	100	7	7.0	7	100.0
2004	105	16	15.2	14	87.5
2005	96	11	11.5	11	100.0
2006	108	8	7.4	8	100.0
2007	75	2	2.7	2	100.0
2008	90	3	3.3	3	100.0
2009	83	9	10.8	8	88.9
2010	74	2	2.7	2	100.0
2011	85	4	4.7	4	100.0
2012	79	3	3.8	3	100.0
2013	64	8	12.5	8	100.0
2014	70	5	7.1	5	100.0
2015	68	8	11.8	7	88.9
2016	80	6	7.5	6	100.0
2017	66	0	0.0	0	0.0
2018	67	0	0.0	0	0.0
2019	57	4	7.0	4	100.0
2020	35	0	0.0	0	0.0
2021	38	1	2.6	1	100.0
Total	1597	111	6.9	100	90.1

* Selected NPEV samples in this study.

**Table 2:** Distribution of NPEVs cases according to age groups, from 2001 to 2021.

Age	NPEV	Total
Year	No.	%
≤1	40	36.0
02-05	47	42.3
06-15	20	18.0
NI *	04	3.7
Total	111	100.0

* NI: not indicated.

**Table 3:** Distribution of NPEVs types detected in patients under 15 years of age with acute AFP in the state of São Paulo, Brazil, from 2001 to 2021.

Species	Type	2001	2002	2003	2004	2005	2006	2007	2008	2009	2010	2011	2012	2013	2014	2015	2016	2017	2018	2019	2020	2021	Total n (%)
*A*	CVA2	1	-	-	-	-	-	-	-	-	-	-	-	-	-	-	-	-	-	-	-	-	1 (1.1)
	CVA3	-	-	-	-	1	-	-	-	-	-	-	-	-	-	-	-	-	-	-	-	-	1 (1.1)
	CVA4	-	-	1	1	-	-	-	-	-	-	-	1	-	-	1	-	-	-	-	-	-	4 (4.4)
	CVA5	-	-	-	1	-	-	-	-	1	-	-	-	-	-	-	-	-	-	-	-	-	2 (2.2)
	CVA6	-	1	-	-	-	-	-	-	-	-	-	-	-	-	-	-	-	-	-	-	-	1 (1.1)
	CVA8	-	-	-	-	-	-	-	-	-	-	-	1	-	-	-	-	-	-	-	-	-	1 (1.1)
	CVA10	-	-	-	-	-	1	-	-	-	-	-	-	-	-	-	-	-	-	-	-	-	1 (1.1)
	CVA16	-	-	-	-	1	-	-	-	-	-	-	-	3	-	1	-	-	-	-	-	-	5 (5.6)
	EV-A71	-	-	-	1	2	1	-	1	-	-	-	-	1	-	2	-	-	-	1	-	-	9 (10.0)
Subtotal		1	1	1	3	4	2	-	1	1	-	-	2	4	-	4	-	-	-	1	-	-	25 (27.8)
*B*	CVB1	-	-	-	-	-	1	-	-	-	-	-	-	-	-	-	-	-	-	-	-	-	1 (1.1)
	CVB2	-	-	1	2	3	1	-	-	1	1	-	-	-	-	-	-	-	-	-	-	-	9 (10.0)
	CVB3	-	-	1	-	-	-	-	-	-	1	-	-	1	-	1	1	-	-	1	-	-	6 (6.7)
	CVB4	-	-	-	-	-	-	-	-	-	-	-	-	-	-	-	1	-	-	-	-	-	1 (1.1)
	CVB5	-	-	1	-	-	1	-	-	-	-	-	1	-	-	-	1	-	-	-	-	-	4 (4.4)
	E1	-	-	-	1	-	-	-	-	-	-	-	-	-	-	-	1	-	-	-	-	1	3 (3.3)
	E3	-	-	-	-	-	-	-	1	-	-	-	-	-	-	-	-	-	-	-	-	-	1 (1.1)
	E6	-	-	-	1	-	-	-	-	2	-	3	-	-	-	-	-	-	-	1	-	-	7 (7.8)
	E7	-	-	-	-	-	-	-	-	-	-	-	-	-	2	-	-	-	-	-	-	-	2 (2.2)
	E9	-	1	-	-	-	-	-	-	-	-	-	-	-	-	-	-	-	-	-	-	-	1 (1.1)
	E11	-	-	-	1	-	-	2	1	2	-	1	-	1	-	1	-	-	-	-	-	-	9 (10.0)
	E13	-	-	-	-	-	1	-	-	-	-	-	-	-	-	-	-	-	-	-	-	-	1 (1.1)
	E14	-	-	1	-	-	-	-	-	-	-	-	-	-	-	-	-	-	-	-	-	-	1 (1.1)
	E16	-	-	-	-	-	1	-	-	-	-	-	-	-	-	-	-	-	-	-	-	-	1 (1.1)
	E18	-	-	-	-	1	-	-	-	-	-	-	-	-	-	-	-	-	-	-	-	-	1 (1.1)
	E25	-	-	-	-	1	-	-	-	-	-	-	-	-	-	-	-	-	-	-	-	-	1 (1.1)
	E30	-	-	-	-	3	-	-	-	-	-	-	-	-	-	-	1	-	-	-	-	-	4 (4.4)
Subtotal		-	1	4	5	8	5	2	2	5	2	4	1	2	2	2	5	-	-	2	-	1	53 (58.9)
*C*	CVA11	-	1	-	-	-	-	-	-	-	-	-	-	-	-	-	-	-	-	-	-	-	1 (1.1)
	CVA13	-	1	-	-	-	-	-	-	-	-	-	-	-	-	-	-	-	-	-	-	-	1 (1.1)
	CVA24	-	-	1	-	-	-	-	-	-	-	-	-	-	-	-	-	-	-	-	-	-	1 (1.1)
	CVA19	-	-	1	-	-	-	-	-	-	-	-	-	-	1	-	-	-	-	1	-	-	3 (3.3)
	EV-C99	-	-	-	1	-	1	-	-	2	-	-	-	-	1	1	-	-	-	-	-	-	6 (6.7)
Subtotal		-	2	2	1	-	1	-	-	2	-	-	-	0	2	1	-	-	-	1	-	-	12 (13.3)
Total		1	4	7	9	12	8	2	3	8	2	4	3	6	4	7	5	-	-	4	-	1	90 (100)

Red color: number of genotypes belonging to species *EV-A*; Blue color: number of genotypes belonging to *EV-B* and Orange color: number of genotypes belonging to *EV-C*.

## Data Availability

The consensus sequence of the viruses analyzed in this study was submitted to the GenBank database under the accession numbers OK605910 to OK605916, OL771248 to OL771253, OR454480, OR498905, OR504975 to OR504997, and OR508976 to OR508985.

## Supplementary Materials

The following supporting information can be downloaded at: https://www.mdpi.com/article/10.3390/v16121875/s1, Table S1: Identification of the samples selected to perform the phylogenetic analysis. Table S2: Paralytic disease progression 60 days post-symptom onset among 100 NPEV-positive patients in Sao Paulo, Brazil, from 2001 to 2021.
