# Supplementary material for: Beyond Poliomyelitis: A 21-Year Study of Non-Polio Enterovirus Genotyping and Its Relevance in Acute Flaccid Paralysis in São Paulo, Brazil
Source: Viruses. 2024 Dec 1;16(12):1875. doi: 10.3390/v16121875 (PMC11680237; doi:10.3390/v16121875)
Supplement: Supplementary file 1 [file viruses-16-01875-s001.zip › Supplementary Table S1.pdf]

Supplementary Table S1a. Identification of the samples selected to perform the phylogenetic analysis.

Isolate name	Accession number	Specie	Genotype	Country	Year	Reference	Diagnostic or I.C.D.
IAL-086/BRA/SP/2001	OR504979		CVA2	Brazil	2001	This study	G37.9
IAL-094/BRA/SP/2005	OR498905		CVA3	Brazil	2005	This study	G61.9
IAL-007/BRA/SP/2003	OR504978		CVA4	Brazil	2003	This study	G83.9
IAL-081/BRA/SP/2009	OR504977		CVA5	Brazil	2009	This study	G83.9
IAL-029/BRA/SP/2002	OR454480		CVA6	Brazil	2002	This study	G61.0
IAL-036/BRA/SP/2012	OR504975		CVA8	Brazil	2012	This study	G98
IAL-090/BRA/SP/2006	OR504976		CVA10	Brazil	2006	This study	G61.0
IAL-059/BRA/SP/2013	OR504980		CVA16	Brazil	2013	This study	G98
IAL-033/BRA/SP/2015	OK605910		EV-A71 C2	Brazil	2015	This study	G04.8
IAL-034/BRA/SP/2008	OK605911		EV-A71 B	Brazil	2008	This study	A08.3
IAL-036/BRA/SP/2013	OK605912		EV-A71 C2	Brazil	2013	This study	G37.9
IAL-069/BRA/SP/2005	OK605913		EV-A71 B	Brazil	2005	This study	G61.0
IAL-120/BRA/SP/2006	OK605914		EV-A71 B	Brazil	2006	This study	N.A.
IAL-041/BRA/SP/2019	OK605915		EV-A71 C1	Brazil	2019	This study	N.A.
IAL-053/BRA/SP/2015	OK605916		EV-A71 C2	Brazil	2015	This study	G99.2
Hyogo1284	LC126145.1		CVA6	Japan	1999-06	Unpublished	N.A.
Kyoto1	AB779614.1	*A*	CVA6	Japan	1999	Unpublished	H.F.M.D.
Hu/BRA/2010/CV-A6/PA	MT491213.1		CVA6	Brazil	2010	Machado et al. 2020[1]	A.G.
SWE05/L0747	GU248448.1		CVA6	Finland	2005	Blomqvist et al. 2010[2]	H.F.M.D.
Gdula	AY421764.1		CVA6	USA	1949	Oberste et al. 2004[3]	N.A.
N.A.	LT577805.1		CVA16	France	2014	Unpublished	N.A.
N.A.	MF093660.1		CVA16	Uruguay	2011	Lizasoain et al. 2018[4]	N.A.
39507/BRA-AP/2009	MH598941.1		CVA16	Brazil	2009	Sousa et al. 2018[5]	H.F.M.D.
48442/BRA-PR/2016	MH598966.1		CVA16	Brazil	2016	Sousa et al. 2018[5]	H.F.M.D.
N.A.	NC_001612.1		CVA16	Finland	1994	Pöyry et al. 1994[6]	N.A.
Hu/MOZ/2016/CV-A16	MT822165.1		CVA16	Mozambique	2016	Unpublished	A.D.
40862/BRA-RJ/2011	MH598953.1		CVA16	Brazil	2011	Sousa et al. 2018[5]	H.F.M.D.
Hu/BRA/2010/CV-A5/PA	MT491211.1		CVA5	Brazil	2010	Unpublished	A.G.
Hu/BRA/2011/CV-A5/PA	MT491212.1		CVA5	Brazil	2011	Unpublished	A.G.

N.A.	KP006021.1		CVA5	China	2013	Unpublished	N.A.
N.A.	AY421763.1		CVA5	USA	1950	Oberste et al. 2004[3]	N.A.
N.A.	JN034206.1		CVA4	Finland	2004	Simonen et al. 2011[7]	N.A
N.A.	KT877425.1		CVA4	Italy	2014	Unpublished	N.A
N.A.	JX009125.1		CVA4	Denmark	2009	Unpublished	N.A.
High Point	AY421762.1		CVA4	USA	1948	Oberste et al. 2004[3]	N.A.
N.A.	KU841452.1		CVA3	Russia	2011	Unpublished	N.A.
26412_CVA3_KGZ_2006	KC879496.1		CVA3	Kyrgyzstan	2006	Lukashev et al. 2014[8]	N.A.
Olson	AY421761.1		CVA3	USA	1948	Oberste et al. 2004[3]	N.A.
Hu/BRA/2011/CV-A10/PA	MT491210.1		CVA10	Brazil	2011	Machado et al. 2020[1]	A.G.
N.A.	KY866439.1		CVA10	Netherlands	2014	Benschop et al. 2017[9]	N.A.
N.A.	KC893470.1		CVA10	Netherlands	2008	Unpublished	N.A.
Kowalik	AY421767.1		CVA10	USA	1950	Oberste et al. 2004[3]	N.A
443-2-YX-YN-2011	AB794105.1		CVA2	China	2011	Unpublished	N.A.
N.A.	JN168999.1		CVA2	China	2009	Xiang et al. 2012[10]	R.T.I.
N.A.	KX021227.1		CVA2	Thailand	2015	Unpublished	H.D.
0112-2006_CV-A2	MT661875.1		CVA2	Uganda	2006	Unpublished	A.F.P.
CVA2-10041-2011	KM816568.1		CVA2	Taiwan	2011	Unpublished	N.A.
Fleetwood	NC_038306.1		CVA2	USA	1947	Oberste et al. 2004[3]	N.A.
N.A.	KC893485.1	*A*	CVA8	Netherlands	2011	Unpublished	N.A.
N.A.	AB848735.1		CVA8	Japan	2013	Unpublished	H.F.M.D.
N.A.	MK111161.1		CVA8	Cyprus	2012	Unpublished	N.A.
N.A.	MK307053.2		CVA8	China	2016	Unpublished	H.F.M.D.
Donovan	AY421766.1		CVA8	USA	1949	Oberste et al. 2004[3]	N.A.
BrCr	U22521.1		EV-A71	USA	1970	Brown et al. 1995[11]	N.A.
GB10/Ab/W/10	HQ676183.1		EV-A71	U. K.	2010	Unpublished	N.A.
N.A.	MW196707.1		EV-A71	Argentina	2017	Lizasoain et al. 2021[12]	Sewage
N.A.	KU645338.1		EV-A71	Ireland	2011	Unpublished	N.A.
N.A.	AB524118.1		EV-A71	Netherlands	1973	Sanden et al. 2010[13]	N.A.

73442-PA-99	AY278249.1		EV-A71	Brazil	1999	Castro et al. 2005[14]	N.A
N.A.	AB524117.1		EV-A71	Netherlands	1976	Sanden et al. 2010[13]	N.A
N.A.	KX372324.1		EV-A71	Thailand	2013	Unpublished	N.A
STU23_DEU10	HG934258.1		EV-A71	Germany	2010	Hassel et al. 2015[15]	H.F.M.D.
CF119026_FRA13	HG934276.1		EV-A71	France	2013	Hassel et al. 2015[15]	H.F.M.D.
N.A.	KY041858.1		EV-A71	Indonesia	2016	Unpublished	H.F.M.D.
6658-COL-94	AF135899.1		EV-A71	Colombia	1994	Brown et al. 2000[16]	Paralysis
C7/Osaka/Japan/97	LC375765.1	*A*	EV-A71	Japan	1997	Shimizu et al. 1999[17]	N.A.
EVA71/Attiki.GR/10.16	MG604327.1		EV-A71	Greece	2016	Unpublished	N.A.
N.A.	MH536836.1		EV-A71	Hungary	2017	Unpublished	N.A.
N.A.	MH256664.1		EV-A71	Switzerland	2016	Tseligka et al. 2018[18]	N.A.
N.A.	MH084321.1		EV-A71	U. K.	2015	Majumdar et al. 2018[19]	Sewage
N.A.	KJ645799.1		EV-A71	Russia	2012	Akhmadishina et al. 2014[20]	A.F.P.
C2-NHRIEV95528-TW	KF306101.1		EV-A71	Taiwan	2012	Unpublished	N.A.
FLU4059	KJ407269.1		EV-A71	Peru	2006	Unpublished	N.A.
IAL-015/BRA/SP/2004	OR504990		CVB2	Brazil	2004	This study	G61.0
IAL-082/BRA/SP/2009	OR504989		CVB2	Brazil	2009	This study	G04.9
IAL-051/BRA/SP/2013	OR504984		CVB3	Brazil	2013	This study	G61.0
IAL-049/BRA/SP/2015	OR504982		CVB3	Brazil	2015	This study	N.A.
IAL-011/BRA/SP/2016	OR504981		CVB3	Brazil	2016	This study	G61.0
IAL-052/BRA/SP/2019	OR504983		CVB3	Brazil	2019	This study	G72.3
IAL-070/BRA/SP/2016	OR504991	*B*	CVB4	Brazil	2016	This study	C72.9
IAL-120/BRA/SP/2003	OR504986		CVB5	Brazil	2003	This study	N.A.
IAL-121/BRA/SP/2006	OR504985		CVB5	Brazil	2006	This study	G61.0
IAL-072/BRA/SP/2012	OR504987		CVB5	Brazil	2012	This study	G61.0
IAL-022/BRA/SP/2016	OR504988		CVB5	Brazil	2016	This study	N.A.
IAL-099/BRA/SP/2004	OR504994		E1	Brazil	2004	This study	G61.0
IAL-037/BRA/SP/2016	OR504995		E1	Brazil	2016	This study	G61.0
IAL-041/BRA/SP/2021	OR504993		E1	Brazil	2021	This study	G61.0
IAL-100/BRA/SP/2008	OR508978		E3	Brazil	2008	This study	G83.9
IAL-079/BRA/SP/2009	OR504996		E6	Brazil	2009	This study	G61.0
IAL-004/BRA/SP/2019	OR504997		E6	Brazil	2019	This study	G04.9

IAL-022/BRA/SP/2014	OR508981		E7	Brazil	2014	This study	N.A.
IAL-032/BRA/SP/2008	OR508980		E11	Brazil	2008	This study	A08.3
IAL-014/BRA/SP/2009	OR508979		E11	Brazil	2009	This study	G61.9
IAL-014/BRA/SP/2003	OR508977		E14	Brazil	2003	This study	G61.0
IAL-048/BRA/SP/2016	OR508976	*B*	E16	Brazil	2016	This study	G62.9
IAL-068/BRA/SP/2016	OR504992		E30	Brazil	2005	This study	N.A.
N.A.	FJ868285.1		CVB2	Australia	2005	Zhou et al. 2009[21]	N.A.
N.A.	AM159196.1		CVB2	France	2003	Andréoletti et al. 2007[22]	A.M.I.
N.A.	AM492491.1		CVB2	France	2000	Unpublished	N.A
N.A.	DQ869817.1		CVB2	France	2006	Nasri et al. 2007[23]	N.A.
N.A.	FJ868285.1		CVB2	Australia	2005	Zhou et al. 2009[21]	N.A.
23.13	KX187377.1		CVB2	Greece	2013	Unpublished	N.A.
Ohio	AF081485.1		CVB2	Germany	N.A.	Unpublished	N.A.
Hu/BRA/2010/CV-B3/PA	MT491224.1		CVB3	Brazil	2010	Machado et al. 2020[1]	A.G.
Hu/BRA/2010/CV-B3/PA	MT491222.1		CVB3	Brazil	2010	Machado et al. 2020[1]	A.G.
03.14	KX187381.1		CVB3	Greece	2014	Unpublished	N.A.
CoxB3/237409.GR	MH544464.1		CVB3	Greece	2013	Unpublished	N.A.
11.14	KX187380.1		CVB3	Greece	2014	Unpublished	Sewage
N.A.	KY865920.1		CVB3	Netherlands	2013	Benschop et al. 2017[9]	Sewage
N.A.	M33854.1		CVB3	N.A.	Prototype	Klump et al. 1990[24]	N.A.
Nancy	JN048468.1		CVB3	USA	1950's	Pan et al. 2011[25]	N.A.
CV-B4/FAMERP_LR236	MN418896.1		CVB4	Brazil	2017	Rocha et al. 2021[26]	N.A.
N.A.	KY433745.1		CVB4	Guinea	2013	Fernandez-Garcia et al. 2017[27]	A.F.P.
B.RD201603	KY861149.1		CVB4	South Africa	2016	Unpublished	Wastewater
J.V.B. Benschoten	X05690.1		CVB4	U.K	Prototype	Jenkins et al. 1987[28]	N.A.
12I10	MN749145.1		CVB5	USA	2015	Unpublished	N.A.
IAL-E1186	KM111284.1		CVB5	Brazil	2012	Unpublished	A.M.
EU13/Mor/08	GU903885.2		CVB5	Morocco	2008	Amdiouni et al. 2012[29]	Wastewater
CoxB5/234763.GR/09.13	MH544447.1		CVB5	Greece	2013	Unpublished	N.A.
NIC007_CYP_05	HF948243.1		CVB5	Cyprus	2005	Henquell et al. 2013[30]	N.A.
Faulkner	AF114383.1		CVB5	Sweden	Prototype	Lindberg et al. 2000[31]	N.A.
N.A.	JN203657.1		E1	India	N.A.	Rao et al. 2012[32]	A.F.P.
N.A.	MW775345.1		E1	Brazil	2014	do Socorro et al. 2021[33]	A.G.
N.A.	KY433701.1	*B*	E1	Senegal	2014	Fernandez et al. 2017[27]	A.F.P.
Farouk; ATCC VR-1038	AF029859.2		E1	N.A.	Prototype	Unpublished	N.A.
Hu/BRA/2010/E3/PA	MT491216.1		E3	Brazil	2010	Machado et al. 2020[1]	A.G.
N.A.	AM492475.1		E3	France	2005	Unpublished	R.T.I.

LR31G7	FJ766334.1		E3	Greece	2005	Kyriakopoulou et al. 2010[34]	E.S.
Morrisey	AY302553.1		E3	USA	1951	Oberste et al. 2004[35]	N.A.
Hu/BRA/2010/E6	MT212621.1		E6	Brazil	2010	Unpublished	A.F.P.
N.A	MH809541.1		E6	Netherlands	2008	Monge et al. 2018[36]	N.A
Hu/BRA/2016/E6	MT212627.1		E6	Brazil	2016	Unpublished	A.F.P.
D'Amori	AY302558.1		E6	USA	1955	Oberste et al. 2004[35]	N.A.
N.A.	JN996505.1		E7	Finland	2001- 07	McLeish et al. 2012[37]	N.A.
N.A.	AM492472.1		E7	France	1999	Unpublished	R.T.I.
N.A.	JN996506.1		E7	Finland	2001- 07	McLeish et al. 201237	N.A.
Wallace	AF465516.1		E7	N.A.	Prototype	Unpublished	N.A.
Hu/E9/IAL-vgf261/2003/BRA	KF182326.1		E9	Brazil	2003	Figueiredo et al. 2014[38]	N.A.
N.A.	MK697681.1		E9	Australia	2013	Stelzer-Braid et al. 2020[39]	N.A.
N.A.	MK086194.1		E9	France	2015	Unpublished	Wastewater
Hill	X84981.1		E9	USA	1953	Zimmermann et al. 1995[40]	N.A.
N.A.	KJ830689.1		E11	Netherlands	2010	Benschop et al. 2015[41]	N.A.
N.A.	MW775346.1		E11	Brazil	2013	do Socorro et al. 2021[33]	A.G.
N.A.	HG793711.1		E11	France	2012	Volle et al. 2014[42]	Meningitis
USA/MI/2016-23031	MH752989.1		E11	USA	2016	Unpublished	N.A.
Silva	AF081326.1		E11	USA	Prototype	Oberste et al. 1999[43]	N.A.
Gregory	X80059.1		E11	USA	1963	Dahllund et al. 1995[44]	N.A.
N.A	AM711098.1		E13	France	2006	Mirand et al. 2008[45]	A.M.
N.A.	AM711059.1		E13	France	2006	Mirand et al. 2008[45]	Meningitis
N.A.	AM236982.1	*B*	E13	France	2005	Mirand et al. 2006[46]	Meningitis
Del Carmen	AY302539.1		E13	USA	1953	Oberste et al. 2004[35]	N.A.
E13_CF235029_FRA06	HF948101.1		E13	France	2006	Henquell et al. 2013[30]	N.A.
N.A.	KU561029.1		E13	Netherlands	2011	de Crom et al. 2016[47]	N.A.
Hu/BRA/2011/E14/PA	MT491220.1		E14	Brazil	2011	Machado et al. 2020[1]	A.G.
N.A.	AB268216.1		E14	China	1997	Bingjun et al. 2008[48]	N.A.
Tow	AY302540.1		E14	USA	1954	Oberste et al. 2004[35]	N.A.
CLI-B3-50-E16	MT641428.1		E16	United Kingdom	2018	Unpublished	N.A.
N.A.	AB598068.1		E16	China	2010	Unpublished	N.A.
N.A.	MK086164.1		E16	France	2014	Unpublished	Wastewater
Harrington	AY302542.1		E16	USA	1951	Oberste et al. 200435	N.A.
N.A.	MF838733.1		E18	Australia	2011	Unpublished	N.A.
N.A.	HG793717.1		E18	France	2012	Volle et al. 2014[42]	Meningitis
N.A.	AM236956.1		E18	France	2005	Mirand et al. 200646	N.A.

Metcalf	AF317694.1		E18	N.A.	Prototype	Andersson et al. 2002[49]	N.A.
N.A.	JX139808.1		E25	Russia	2010	Unpublished	A.G.
N.A.	KJ472876.1		E25	Kenya	2008	Unpublished	N.A.
N.A.	AM711106.1		E25	France	2006	Mirand et al. 2008[45]	Meningitis
JV-4	AY302549.1		E25	USA	1957	Oberste et al. 2004[35]	N.A.
N.A.	AJ430700.1		E30	France	1996	Bailly et al. 2002[50]	Meningitis
Hu/BRA/2016/E30/PR_49534	MK570363.1		E30	Brazil	2016	Ramalho et al. 2019[51]	A.M.
N.A.	EU678967.1		E30	Brazil	2005	Unpublished	Meningitis
N.A.	HQ152909.1		E30	Brazil	2005	dos Santos el at. 2011[52]	Meningitis
N.A.	HQ152912.1		E30	Brazil	2005	dos Santos el at. 201152	Meningitis
N.A.	MK410128.1		E30	Argentina	2001	Lema et al. 2019[53]	Meningitis
Giles	AF081342.1		E30	N.A.	N.A.	Oberste et al. 199943	N.A.
IAL-009/BRA/SP/2002	OR508983		CVA11	Brazil	2002	This study	G83.9
IAL-037/BRA/SP/2002	OR508984		CVA13	Brazil	2002	This study	G83.9
IAL-027/BRA/SP/2019	OR508985		CVA19	Brazil	2019	This study	N.A.
IAL-015/BRA/SP/2003	OR508982		CVA24 IV	Brazil	2003	This study	G61.0
IAL-005/BRA/SP/2009	OL771248		EV-C99	Brazil	2009	This study	G04.9
IAL-006/BRA/SP/2006	OL771249		EV-C99	Brazil	2006	This study	G04.9
IAL-015/BRA/SP/2014	OL771250		EV-C99	Brazil	2014	This study	G05.8
IAL-024/BRA/SP/2009	OL771251		EV-C99	Brazil	2009	This study	G61.0
IAL-051/BRA/SP/2015	OL771252		EV-C99	Brazil	2015	This study	N.A.
IAL-111/BRA/SP/2004	OL771253		EV-C99	Brazil	2004	This study	G61.0
Human/EV-C99/BRA/TO-16/BRA/2013	MK689071.1		EV-C99	Brazil	2013	Luchs et al. 2019[54]	A.G.
Hu/BRA/2010/EV99/PA	MT491239.1		EV-C99	Brazil	2010	Machado et al. 2020[1]	A.G.
N.A	KF129039.1		EV-C99	Finland	2007	Smura et al. 2014[55]	N.A.
03-1143	MG793387.1		EV-C99	Malawi	2003	Brouwer et al. 2018[56]	N.A.
03-1247	MG793390.1		EV-C99	Malawi	2003	Brouwer et al. 2018[56]	N.A.
03-4076	MG793393.1		EV-C99	Malawi	2003	Brouwer et al. 201856	N.A.
N.A	MF093682.1		EV-C99	Uruguay	2011	Lizasoain et al. 20184	E.S.
12-008-2_C99	MH144606.1		EV-C99	India	2012	Unpublished	N.A.
MCF716NGR19	MN563088.1		EV-C99	Nigeria	2019	Unpublished	A.F.P.
USA-Ok85-10627	EF015012.1	*C*	EV-C99	USA	1985	Brown et al. 2009[57]	N.A.
N.A	KJ857508.1		EV-C99	China	2011	Tao et al. 2014[58]	N.A.
K292/YN/CHN/2013	KT946713.1		EV-C99	China	2013	Unpublished	N.A.
N.A	MK086465.1		CVA11	France	2014	Unpublished	Wastewater
Belgium-1	AF499636.1		CVA11	Belgium	1951	Brown et al. 2003[59]	N.A.
N.A	GU983178.1		CVA24	Brazil	2003	Tavares et al. 2011[60]	A.H.C.

N.A	GU983171.1	CVA24	Brazil	2003	Tavares et al. 2011[60]	A.H.C.
N.A	GU983170.1	CVA24	Brazil	2003	Tavares et al. 2011[60]	A.H.C.
N.A	GU983189.1	CVA24	Brazil	2003	Tavares et al. 201160	A.H.C.
N.A	D90457.1	CVA24	Singapore	1970's	Supanaranond et al. 1992[61]	A.H.C.
N.A	MK086488.1	CVA19	France	2014	Unpublished	Wastewater
Hu/MOZ/2015/CV-A19/ZB 0812	MT822164.1	CVA19	Mozambique	2015	Unpublished	A.D.
N.A	MN812173.1	CVA19	Ghana	2012	Di Cristanziano et al. 2020[62]	N.A.
N.A	KX932039.1	CVA19	Switzerland	2015	Unpublished	N.A.
8663	AF499641.1	CVA19	Japan	1952	Brown et al. 2003[59]	N.A.
Hu/BRA/2007/CV-A13	MT271235.1	CVA13	Brazil	2007	Unpublished	N.A.
Hu/BRA/2005/CV-A13	MT271232.1	CVA13	Brazil	2005	Unpublished	N.A.
Hu/BRA/2006 CV-A13	MT271233.1	CVA13	Brazil	2006	Unpublished	N.A.
N.A	KJ933342.1	CVA13	China	2013	Unpublished	N.A.
Flores	AF499637.1	CVA13	Mexico	1952	Brown et al. 2003[59]	N.A.

N.A – Not Available; H.F.M.D. Hand, Foot and Mouth Disease; A.G. Acute gastroenteritis; A.D. Acute diarrhea; R.T.I. Respiratory tract infection; H.D. Herpangina disease; A.F.P. Acute flaccid paralysis; A.M.I. Acute myocardial infarction; A.M. Aseptic meningitis; E.S. Environmental samples; A.H.C. Acute Hemorrhagic Conjunctivitis.

Supplementary Table S1b - International Classification of Diseases.

ICD	Diagnostic
G37.9	Demyelinating disease of central nervous system, unspecified
G61.9	Inflammatory polyneuropathy, unspecified
G83.9	Paralytic syndrome, unspecified
G61.0	Guillain-Barré syndrome
G98	Other disorders of nervous system, not elsewhere classified
G04.8	Other encephalitis, myelitis and encephalomyelitis
A08.3	Other viral enteritis
G99.2	Myelopathy in diseases classified elsewhere
G05.8	Encephalitis, myelitis and encephalomyelitis in other diseases classified elsewhere
G04.9	Encephalitis, myelitis and encephalomyelitis, unspecified
C72.9	Central nervous system, unspecified
G62.9	Polyneuropathy, unspecified
G72.3	Periodic paralysis
G71.2	Congenital myopathies

https://icd.who.int/browse10/2019/en

REFERENCES

1 Machado RS, de Sousa IP Jr, Monteiro JC, Ferreira JL, Dos Santos Alves JC, Tavares FN. Detection and identification of enteroviruses circulating in children with acute gastroenteritis in Pará State, Northern Brazil (2010-2011). Virol J. 2020 Oct 16;17(1):156. doi: 10.1186/s12985-020-01431-w. PMID: 33066782; PMCID: PMC7565352.

2 Blomqvist S, Klemola P, Kaijalainen S, Paananen A, Simonen ML, Vuorinen T, Roivainen M. Co-circulation of coxsackieviruses A6 and A10 in hand, foot and mouth disease outbreak in Finland. J Clin Virol. 2010 May;48(1):49-54. doi: 10.1016/j.jcv.2010.02.002. Epub 2010 Feb 26. PMID: 20189452.

3 Oberste MS, Peñaranda S, Maher K, Pallansch MA. Complete genome sequences of all members of the species Human enterovirus A. J Gen Virol. 2004 Jun;85(Pt 6):1597-1607. doi: 10.1099/vir.0.79789-0. PMID: 15166444.

4 Lizasoain A, Burlandy FM, Victoria M, Tort LFL, da Silva EE, Colina R. An Environmental Surveillance in Uruguay Reveals the Presence of Highly Divergent Types of Human Enterovirus Species C and a High Frequency of Species A and B Types. Food Environ Virol. 2018 Dec;10(4):343-352. doi: 10.1007/s12560-018-9351-7. Epub 2018 Jun 16. PMID: 29907902.

5 Sousa IP Jr, Burlandy FM, Costa EV, Tavares FN, da Silva EE. Enteroviruses associated with hand, foot, and mouth disease in Brazil. J Infect. 2018 Nov;77(5):448-454. doi: 10.1016/j.jinf.2018.08.012. Epub 2018 Aug 24. PMID: 30149028.

6 Pöyry T, Hyypiä T, Horsnell C, Kinnunen L, Hovi T, Stanway G. Molecular analysis of coxsackievirus A16 reveals a new genetic group of enteroviruses. Virology. 1994 Aug 1;202(2):982-7. doi: 10.1006/viro.1994.1423. PMID: 8030260.

7 Simonen-Tikka ML, Pflueger M, Klemola P, Savolainen-Kopra C, Smura T, Hummel S, Kaijalainen S, Nuutila K, Natri O, Roivainen M, Ziegler AG. Human enterovirus infections in children at increased risk for type 1 diabetes: the Babydiet study. Diabetologia. 2011 Dec;54(12):2995-3002. doi: 10.1007/s00125-011-2305-3. Epub 2011 Sep 20. PMID: 21932150.

8 Lukashev AN, Shumilina EY, Belalov IS, Ivanova OE, Eremeeva TP, Reznik VI, Trotsenko OE, Drexler JF, Drosten C. Recombination strategies and evolutionary dynamics of the Human enterovirus A global gene pool. J Gen Virol. 2014 Apr;95(Pt 4):868-873. doi: 10.1099/vir.0.060004-0. Epub 2014 Jan 14. PMID: 24425417.

9 Benschop KSM, van der Avoort HG, Jusic E, Vennema H, van Binnendijk R, Duizer E. Polio and Measles Down the Drain: Environmental Enterovirus Surveillance in the Netherlands, 2005 to 2015. Appl Environ Microbiol. 2017 Jun 16;83(13):e00558-17. doi: 10.1128/AEM.00558-17. PMID: 28432101; PMCID: PMC5478994.

10 Xiang Z, Gonzalez R, Wang Z, Ren L, Xiao Y, Li J, Li Y, Vernet G, Paranhos-Baccalà G, Jin Q, Wang J. Coxsackievirus A21, enterovirus 68, and acute respiratory tract infection, China. Emerg Infect Dis. 2012 May;18(5):821-4. doi: 10.3201/eid1805.111376. PMID: 22516379; PMCID: PMC3358056.

11 Brown BA, Pallansch MA. Complete nucleotide sequence of enterovirus 71 is distinct from poliovirus. Virus Res. 1995 Dec;39(2-3):195-205. doi: 10.1016/0168-1702(95)00087-9. PMID: 8837884.

12 Lizasoain A, Mir D, Salvo M, Bortagaray V, Masachessi G, Farías A, Rodríguez-Osorio N, Nates S, Victoria M, Colina R. First evidence of enterovirus A71 and echovirus 30 in Uruguay and genetic relationship with strains circulating in the South American region. PLoS One. 2021 Aug 12;16(8):e0255846.

doi: 10.1371/journal.pone.0255846. PMID: 34383835; PMCID: PMC8360592.

13 van der Sanden S, van der Avoort H, Lemey P, Uslu G, Koopmans M. Evolutionary trajectory of the VP1 gene of human enterovirus 71 genogroup B and C viruses. J Gen Virol. 2010 Aug;91(Pt 8):1949-1958. doi: 10.1099/vir.0.019695-0. Epub 2010 Apr 7. PMID: 20375223.

14 Castro CM, Cruz AC, Silva EE, Gomes Mde L. Molecular and seroepidemiologic studies of Enterovirus 71 infection in the State of Para, Brazil. Rev Inst Med Trop Sao Paulo. 2005 Mar-Apr;47(2):65-71. doi: 10.1590/s0036-46652005000200002. Epub 2005 May 4. PMID: 15880216.

15 Hassel C, Mirand A, Lukashev A, TerletskaiaLadwig E, Farkas A, Schuffenecker I, Diedrich S, Huemer HP, Archimbaud C, Peigue-Lafeuille H, Henquell C, Bailly JL. Transmission patterns of human enterovirus 71 to, from and among European countries, 2003 to 2013. Euro Surveill. 2015;20(34):30005. doi: 10.2807/1560-7917.ES.2015.20.34.30005. PMID: 26530407.

16 Brown BA, Oberste MS, Alexander JP Jr, Kennett ML, Pallansch MA. Molecular epidemiology and evolution of enterovirus 71 strains isolated from 1970 to 1998. J Virol. 1999 Dec;73(12):9969-75. doi: 10.1128/JVI.73.12.9969-9975.1999. Erratum in: J Virol. 2000 Dec;74(24):12003. PMID: 10559310; PMCID: PMC113047.

17 Shimizu H, Utama A, Yoshii K, Yoshida H, Yoneyama T, Sinniah M, Yusof MA, Okuno Y, Okabe N, Shih SR, Chen HY, Wang GR, Kao CL, Chang KS, Miyamura T, Hagiwara A. Enterovirus 71 from fatal and nonfatal cases of hand, foot and mouth disease epidemics in Malaysia, Japan and Taiwan in 1997-1998. Jpn J Infect Dis. 1999 Feb;52(1):12-5. PMID: 10808253.

18 Tseligka ED, Sobo K, Stoppini L, Cagno V, Abdul F, Piuz I, Meylan P, Huang S, Constant S, Tapparel C. A VP1 mutation acquired during an enterovirus 71 disseminated infection confers heparan sulfate binding ability and modulates ex vivo tropism. PLoS Pathog. 2018 Aug 3;14(8):e1007190. doi: 10.1371/journal.ppat.1007190. PMID: 30075025; PMCID: PMC6093697.

19 Majumdar M, Sharif S, Klapsa D, Wilton T, Alam MM, Fernandez-Garcia MD, Rehman L, Mujtaba G, McAllister G, Harvala H, Templeton K, Mee ET, Asghar H, Ndiaye K, Minor PD, Martin J. Environmental Surveillance Reveals Complex Enterovirus Circulation Patterns in Human Populations. Open Forum Infect Dis. 2018 Oct 1;5(10):ofy250. doi: 10.1093/ofid/ofy250. PMID: 30377626; PMCID: PMC6201154.

20 Akhmadishina LV, Eremeeva TP, Trotsenko OE, Ivanova OE, Mikhailov MI, Lukashev AN. Seroepidemiology and molecular epidemiology of enterovirus 71 in Russia. PLoS One. 2014 May 12;9(5):e97404. doi: 10.1371/journal.pone.0097404. PMID: 24819617; PMCID: PMC4018281.

21 Zhou F, Kong F, McPhie K, Ratnamohan M, Donovan L, Zeng F, Gilbert GL, Dwyer DE. Identification of 20 common human enterovirus serotypes by use of a reverse transcription-PCR-based reverse line blot hybridization assay. J Clin Microbiol. 2009 Sep;47(9):2737-43. doi: 10.1128/JCM.00823-09. Epub 2009 Jul 1. PMID: 19571022; PMCID: PMC2738090.

22 Andréoletti L, Ventéo L, Douche-Aourik F, Canas F, Lorin de la Grandmaison G, Jacques J, Moret H, Jovenin N, Mosnier JF, Matta M, Duband S, Pluot M, Pozzetto B, Bourlet T. Active Coxsackieviral B infection is associated with disruption of dystrophin in endomyocardial tissue of patients who died suddenly of acute myocardial infarction. J Am Coll Cardiol. 2007 Dec 4;50(23):2207-14. doi: 10.1016/j.jacc.2007.07.080. Epub 2007 Nov 19. PMID: 18061067.

23 Nasri D, Bouslama L, Omar S, Saoudin H, Bourlet T, Aouni M, Pozzetto B, Pillet S. Typing of human enterovirus by partial sequencing of VP2. J Clin Microbiol. 2007 Aug;45(8):2370-9. doi: 10.1128/JCM.00093-07. Epub 2007 May 30. PMID: 17537940; PMCID: PMC1951248.

24 Klump WM, Bergmann I, Müller BC, Ameis D, Kandolf R. Complete nucleotide sequence of infectious Coxsackievirus B3 cDNA: two initial 5' uridine residues are regained during plus-strand RNA synthesis. J Virol. 1990 Apr;64(4):1573-83. doi: 10.1128/JVI.64.4.1573-1583.1990. PMID: 2157045; PMCID: PMC249292**.**

25 Pan J, Narayanan B, Shah S, Yoder JD, Cifuente JO, Hafenstein S, Bergelson JM. Single amino acid changes in the virus capsid permit coxsackievirus B3 to bind decay-accelerating factor. J Virol. 2011 Jul;85(14):7436-43. doi: 10.1128/JVI.00503-11. Epub 2011 May 11. PMID: 21561916; PMCID: PMC3126562.

26 Rocha LCD, Estofolete CF, Milhim BHGA, Augusto MT, Zini N, Silva GCDD, Ferraz-Junior HC, Brienze VMS, Liso E, Cunha MS, Sabino EC, da Costa AC, Nogueira ML, Luchs A, Terzian ACB. Enteric viruses circulating in undiagnosed central nervous system infections at tertiary hospital in São José do Rio Preto, São Paulo, Brazil. J Med Virol. 2021 Jun;93(6):3539-3548. doi: 10.1002/jmv.26216. Epub 2020 Jul 11. PMID: 32579291.

27 Fernandez-Garcia MD, Kebe O, Fall AD, Ndiaye K. Identification and molecular characterization of non-polio enteroviruses from children with acute flaccid paralysis in West Africa, 2013-2014. Sci Rep. 2017 Jun 19;7(1):3808. doi: 10.1038/s41598-017-03835-1. PMID: 28630462; PMCID: PMC5476622.

28 Jenkins O, Booth JD, Minor PD, Almond JW. The complete nucleotide sequence of coxsackievirus B4 and its comparison to other members of the Picornaviridae. J Gen Virol. 1987 Jul;68 (Pt 7):1835-48. doi: 10.1099/0022-1317-68-7-1835. PMID: 3037008**.**

29 Amdiouni H, Faouzi A, Fariat N, Hassar M, Soukri A, Nourlil J. Detection and molecular identification of human adenoviruses and enteroviruses in wastewater from Morocco. Lett Appl Microbiol. 2012 Apr;54(4):359-66. doi: 10.1111/j.1472-765X.2012.03220.x. Epub 2012 Mar 6. PMID: 22324352.

30 Henquell C, Mirand A, Richter J, Schuffenecker I, Böttiger B, Diedrich S, Terletskaia-Ladwig E, Christodoulou C, Peigue-Lafeuille H, Bailly JL. Phylogenetic patterns of human coxsackievirus B5 arise from population dynamics between two genogroups and reveal evolutionary factors of molecular adaptation and transmission. J Virol. 2013 Nov;87(22):12249-59. doi: 10.1128/JVI.02075-13. Epub 2013 Sep 4. PMID: 24006446; PMCID: PMC3807918.

31 Lindberg AM, Polacek C. Molecular analysis of the prototype coxsackievirus B5 genome. Arch Virol. 2000;145(2):205-21. doi: 10.1007/s007050050019. PMID: 10752549**.**

32 Rao CD, Yergolkar P, Shankarappa KS. Antigenic diversity of enteroviruses associated with nonpolio acute flaccid paralysis, India, 2007-2009. Emerg Infect Dis. 2012 Nov;18(11):1833-40. doi: 10.3201/eid1811.111457. PMID: 23092622; PMCID: PMC3559176.

33 do Socorro Fôro Ramos E, Rosa UA, de Oliveira Ribeiro G, Villanova F, de Pádua Milagres FA, Brustulin R, Dos Santos Morais V, Bertanhe M, Marcatti R, Araújo ELL, Witkin SS, Delwart E, Luchs A, da Costa AC, Leal É. High Heterogeneity of Echoviruses in Brazilian Children with Acute Gastroenteritis. Viruses. 2021 Mar 31;13(4):595. doi: 10.3390/v13040595. PMID: 33807396; PMCID: PMC8067319.

34 Kyriakopoulou Z, Dedepsidis E, Pliaka V, Tsakogiannis D, Pratti A, Levidiotou-Stefanou S, Markoulatos P. Full-genome sequence analysis of a multirecombinant echovirus 3 strain isolated from sewage in Greece. J Clin Microbiol. 2010 May;48(5):1513-9. doi: 10.1128/JCM.00475-09. Epub 2010 Feb 3. PMID: 20129960; PMCID: PMC2863879.

35 Oberste MS, Maher K, Pallansch MA. Evidence for frequent recombination within species human enterovirus B based on complete genomic sequences of all thirty-seven serotypes. J Virol. 2004 Jan;78(2):855-67. doi: 10.1128/jvi.78.2.855-867.2004. PMID: 14694117; PMCID: PMC368751.

36 Monge S, Benschop K, Soetens L, Pijnacker R, Hahné S, Wallinga J, Duizer E. Echovirus type 6 transmission clusters and the role of environmental

surveillance in early warning, the Netherlands, 2007 to 2016. Euro Surveill. 2018 Nov;23(45):1800288. doi: 10.2807/1560-7917.ES.2018.23.45.1800288. PMID: 30424830; PMCID: PMC6234528.

37 McLeish NJ, Williams ÇH, Kaloudas D, Roivainen MM, Stanway G. Symmetry-related clustering of positive charges is a common mechanism for heparan sulfate binding in enteroviruses. J Virol. 2012 Oct;86(20):11163-70. doi: 10.1128/JVI.00640-12. Epub 2012 Aug 1. PMID: 22855495; PMCID: PMC3457184.

38 Figueiredo CA, Luchs A, Russo DH, de Cassia Compagnoli Carmona R, Afonso AM, de Oliveira MI, Curti SP, de Moraes JC, Toscano CM, Ciccone FH, Timenetsky Mdo C. Rubella virus genotype 1G and echovirus 9 as etiologic agents of exanthematous diseases in Brazil: insights from phylogenetic analysis. Arch Virol. 2014 Jun;159(6):1445-51. doi: 10.1007/s00705-013-1935-9. Epub 2013 Dec 11. PMID: 24327091.

39 Stelzer-Braid S, Wynn M, Chatoor R, Scotch M, Ramachandran V, Teoh HL, Farrar MA, Sampaio H, Andrews PI, Craig ME, MacIntyre CR, Varadhan H, Kesson A, Britton PN, Newcombe J, Rawlinson WD. Next generation sequencing of human enterovirus strains from an outbreak of enterovirus A71 shows applicability to outbreak investigations. J Clin Virol. 2020 Jan;122:104216. doi: 10.1016/j.jcv.2019.104216. Epub 2019 Nov 17. PMID: 31790967; PMCID: PMC7384352.

40 Zimmermann H, Eggers HJ, Zimmermann A, Kraus W, Nelsen-Salz B. Complete nucleotide sequence and biological properties of an infectious clone of prototype echovirus 9. Virus Res. 1995 Dec;39(2-3):311-9. doi: 10.1016/0168-1702(95)00078-x. PMID: 8837893.

41 Benschop KSM, Wildenbeest JG, Koen G, Minnaar RP, van Hemert FJ, Westerhuis BM, Pajkrt D, van den Broek PJ, Vossen ACTM, Wolthers KC. Genetic and antigenic structural characterization for resistance of echovirus 11 to pleconaril in an immunocompromised patient. J Gen Virol. 2015 Mar;96(Pt 3):571-579. doi: 10.1099/vir.0.069773-0. Epub 2014 Nov 13. PMID: 25395595.

42 Volle R, Bailly JL, Mirand A, Pereira B, Marque-Juillet S, Chambon M, Regagnon C, Brebion A, Henquell C, Peigue-Lafeuille H, Archimbaud C. Variations in cerebrospinal fluid viral loads among enterovirus genotypes in patients hospitalized with laboratory-confirmed meningitis due to enterovirus. J Infect Dis. 2014 Aug 15;210(4):576-84. doi: 10.1093/infdis/jiu178. Epub 2014 Mar 20. PMID: 24652491.

43 Oberste MS, Maher K, Kilpatrick DR, Pallansch MA. Molecular evolution of the human enteroviruses: correlation of serotype with VP1 sequence and application to picornavirus classification. J Virol. 1999 Mar;73(3):1941-8. doi: 10.1128/JVI.73.3.1941-1948.1999. PMID: 9971773; PMCID: PMC104435.

44 Dahllund L, Nissinen L, Pulli T, Hyttinen VP, Stanway G, Hyypiä T. The genome of echovirus 11. Virus Res. 1995 Feb;35(2):215-22. doi: 10.1016/0168-1702(94)00104-k. PMID: 7762294.

45 Mirand A, Henquell C, Archimbaud C, Chambon M, Charbonne F, Peigue-Lafeuille H, Bailly JL. Prospective identification of enteroviruses involved in meningitis in 2006 through direct genotyping in cerebrospinal fluid. J Clin Microbiol. 2008 Jan;46(1):87-96. doi: 10.1128/JCM.01020-07. Epub 2007 Oct 31. PMID: 17977989; PMCID: PMC2224282.

46 Mirand A, Archimbaud C, Henquell C, Michel Y, Chambon M, Peigue-Lafeuille H, Bailly JL. Prospective identification of HEV-B enteroviruses during the 2005 outbreak. J Med Virol. 2006 Dec;78(12):1624-34. doi: 10.1002/jmv.20747. PMID: 17063526.

47 de Crom SC, Rossen JW, de Moor RA, Veldkamp EJ, van Furth AM, Obihara CC. Prospective assessment of clinical symptoms associated with enterovirus and parechovirus genotypes in a multicenter study in Dutch children. J Clin Virol. 2016 Apr;77:15-20. doi: 10.1016/j.jcv.2016.01.014. Epub 2016

Feb 2. PMID: 26875098.

48 Bingjun T, Yoshida H, Yan W, Lin L, Tsuji T, Shimizu H, Miyamura T. Molecular typing and epidemiology of non-polio enteroviruses isolated from Yunnan Province, the People's Republic of China. J Med Virol. 2008 Apr;80(4):670-9. doi: 10.1002/jmv.21122. PMID: 18297723.

49 Andersson P, Edman K, Lindberg AM. Molecular analysis of the echovirus 18 prototype: evidence of interserotypic recombination with echovirus 9. Virus Res. 2002 Apr 23;85(1):71-83. doi: 10.1016/s0168-1702(02)00019-9. Erratum in: Virus Res. 2003 Feb;91(2):261. PMID: 11955640.

50 Bailly JL, Brosson D, Archimbaud C, Chambon M, Henquell C, Peigue-Lafeuille H. Genetic diversity of echovirus 30 during a meningitis outbreak, demonstrated by direct molecular typing from cerebrospinal fluid. J Med Virol. 2002 Dec;68(4):558-67. doi: 10.1002/jmv.10235. PMID: 12376964.

51 Ramalho E, Sousa I Jr, Burlandy F, Costa E, Dias A, Serrano R, Oliveira M, Lopes R, Debur M, Burger M, Riediger I, Oliveira ML, Nascimento O, da Silva EE. Identification and Phylogenetic Characterization of Human Enteroviruses Isolated from Cases of Aseptic Meningitis in Brazil, 2013-2017. Viruses. 2019 Jul 29;11(8):690. doi: 10.3390/v11080690. PMID: 31362357; PMCID: PMC6723535.

52 dos Santos GP, da Costa EV, Tavares FN, da Costa LJ, da Silva EE. Genetic diversity of Echovirus 30 involved in aseptic meningitis cases in Brazil (1998-2008). J Med Virol. 2011 Dec;83(12):2164-71. doi: 10.1002/jmv.22235. PMID: 22012725.

53 Lema C, Torres C, Van der Sanden S, Cisterna D, Freire MC, Gómez RM. Global phylodynamics of Echovirus 30 revealed differential behavior among viral lineages. Virology. 2019 May;531:79-92. doi: 10.1016/j.virol.2019.02.012. Epub 2019 Feb 18. PMID: 30856485.

54 Luchs A, Leal E, Tardy K, Milagres FAP, Komninakis SV, Brustulin R, Teles MDAR, Lobato MCABS, das Chagas RT, Abrão MFNDS, Soares CVDA, Deng X, Delwart E, Sabino EC, da Costa AC. The rare enterovirus c99 and echovirus 29 strains in Brazil: potential risks associated to silent circulation. Mem Inst Oswaldo Cruz. 2019;114:e190160. doi: 10.1590/0074-02760190160. Epub 2019 Aug 12. PMID: 31411312; PMCID: PMC6690645.

55 Smura T, Blomqvist S, Vuorinen T, Ivanova O, Samoilovich E, Al-Hello H, Savolainen-Kopra C, Hovi T, Roivainen M. The evolution of Vp1 gene in enterovirus C species sub-group that contains types CVA-21, CVA-24, EV-C95, EV-C96 and EV-C99. PLoS One. 2014 Apr 2;9(4):e93737. doi: 10.1371/journal.pone.0093737. PMID: 24695547; PMCID: PMC3973639.

56 Brouwer L, van der Sanden SMG, Calis JCJ, Bruning AHL, Wang S, Wildenbeest JG, Rebers SPH, Phiri KS, Westerhuis BM, van Hensbroek MB, Pajkrt D, Wolthers KC. High frequency of Polio-like Enterovirus C strains with differential clustering of CVA-13 and EV-C99 subgenotypes in a cohort of Malawian children. Arch Virol. 2018 Oct;163(10):2645-2653. doi: 10.1007/s00705-018-3878-7. Epub 2018 May 28. PMID: 29808442; PMCID: PMC6132918.

57 Brown BA, Maher K, Flemister MR, Naraghi-Arani P, Uddin M, Oberste MS, Pallansch MA. Resolving ambiguities in genetic typing of human enterovirus species C clinical isolates and identification of enterovirus 96, 99 and 102. J Gen Virol. 2009 Jul;90(Pt 7):1713-1723. doi: 10.1099/vir.0.008540-0. Epub 2009 Mar 4. PMID: 19264596.

58 Tao Z, Yuan Q, Lin X, Wang S, Liu Y, Ji F, Xiong P, Cui N, Song L, Wang M, Xu A. Molecular characterization of enteroviruses including a new type EV-C99 isolated from Xinjiang students in Shandong, China in 2011. Sci Rep. 2014 Oct 9;4:6564. doi: 10.1038/srep06564. PMID: 25298041; PMCID: PMC4190507.

59 Brown B, Oberste MS, Maher K, Pallansch MA. Complete genomic sequencing shows that polioviruses and members of human enterovirus species C

are closely related in the noncapsid coding region. J Virol. 2003 Aug;77(16):8973-84. doi: 10.1128/jvi.77.16.8973-8984.2003. PMID: 12885914; PMCID: PMC167246.

60 Tavares FN, Campos Rde M, Burlandy FM, Fontella R, de Melo MM, da Costa EV, da Silva EE. Molecular characterization and phylogenetic study of coxsackievirus A24v causing outbreaks of acute hemorrhagic conjunctivitis (AHC) in Brazil. PLoS One. 2011;6(8):e23206. doi: 10.1371/journal.pone.0023206. Epub 2011 Aug 16. PMID: 21858030; PMCID: PMC3156732.

61 Supanaranond K, Takeda N, Yamazaki S. The complete nucleotide sequence of a variant of Coxsackievirus A24, an agent causing acute hemorrhagic conjunctivitis. Virus Genes. 1992 Apr;6(2):149-58. doi: 10.1007/BF01703064. PMID: 1317075.

62 Di Cristanziano V, Weimer K, Böttcher S, Sarfo FS, Dompreh A, Cesar LG, Knops E, Heger E, Wirtz M, Kaiser R, Norman B, Phillips RO, Feldt T, Eberhardt KA. Molecular Characterization and Clinical Description of Non-Polio Enteroviruses Detected in Stool Samples from HIV-Positive and HIV-Negative Adults in Ghana. Viruses. 2020 Feb 16;12(2):221. doi: 10.3390/v12020221. PMID: 32079128; PMCID: PMC7077198.
