# Supplementary material for: Beyond Poliomyelitis: A 21-Year Study of Non-Polio Enterovirus Genotyping and Its Relevance in Acute Flaccid Paralysis in São Paulo, Brazil
Source: Viruses. 2024 Dec 1;16(12):1875. doi: 10.3390/v16121875 (PMC11680237; doi:10.3390/v16121875)
Supplement: Supplementary file 1 [file viruses-16-01875-s001.zip › Supplementary Table S2.pdf]

Supplementary Table S2. Paralytic disease progression 60 days post-symptom onset among 100 NPEV-positive patients, in São Paulo, Brazil, during 2001-2021.

Case no.	Gender	Year	Age		Onset of AFP	Disease evolution	Species NPEV	Genotype	Initial clinical Diagnosis	ICD
1	F	2001	9	Months	2001-09-09	Sequelae	A	CVA2	Acute flaccid myelitis	G37.3
2	M	2002	3	Years	2002-02-21	Recovered	C	CVA11	Unspecified paralytic syndrome	G83.9
3	M	2002	2	Years	2002-05-24	Sequelae	A	CVA6	Guillain-Barré syndrome	G61.0
4	F	2002	4	Years	2002-05-24	Sequelae	B	E9	Unspecified paralytic syndrome	G83.9
5	F	2002	9	Years	2002-05-19	Sequelae	-	NTEV	Unspecified paralytic syndrome	G83.9
6	F	2002	1	Year	2002-04-12	Recovered	C	CVA13	Unspecified paralytic syndrome	G83.9
7	M	2002	3	Years	2002-08-02	Recovered	-	NTEV	Guillain-Barré syndrome	G61.0
8	M	2003	1	Year	2003-01-23	Recovered	A	CVA4	Unspecified paralytic syndrome	G83.9
9	F	2003	4	Years	2003-04-01	Sequelae	B	E14	Guillain-Barré syndrome	G61.0
10	M	2003	7	Years	2003-04-02	Sequelae	C	CVA24 IV	Guillain-Barré syndrome	G61.0
11	M	2003	4	Years	2003-04-20	Recovered	C	CVA19	Guillain-Barré syndrome	G61.0
12	F	2003	1	Year	2003-06-14	Sequelae	B	CVB3	Unspecified paralytic syndrome	G83.9
13	M	2003	1	Year	2003-11-24	NI	B	CVB5	NI	NI
14	F	2003	11	Years	2003-08-28	NI	B	CVB2	NI	NI
15	M	2004	1	Year	2004-02-04	Recovered	A	CVA5	Stroke	I64
16	M	2004	2	Years	2004-02-20	Recovered	B	CVB2	Guillain-Barré syndrome	G61.0
17	F	2004	4	Years	2004-03-03	Recovered	B	CVB2	Guillain-Barré syndrome	G61.0
18	F	2004	NI	NI	2004-03-10	Recovered	-	NTEV	Encephalitis or meningoencephalitis	G04.2
19	M	2004	10	Years	2004-03-10	NI	-	NTEV	Guillain-Barré syndrome	G61.0
20	M	2004	4	Years	2004-03-07	Recovered	-	NTEV	Unspecified paralytic syndrome	G83.9
21	M	2004	NI	NI	2004-03-09	Recovered	-	NTEV	Guillain-Barré syndrome	G61.0
22	F	2004	9	Years	2004-05-05	NI	B	E6	Unspecified paralytic syndrome	G83.9
23	F	2004	5	Years	2004-05-31	NI	B	E11	Guillain-Barré syndrome	G61.0
24	M	2004	2	Years	2004-06-17	Sequelae	-	NTEV	Stroke	I64
25	F	2004	NI	NI	2004-06-26	Recovered	A	CVA4	Encephalitis or meningoencephalitis	G04.2
26	M	2004	1	Year	2004-09-29	NI	A	EV-A71 C1	Neurovirus	A89
27	M	2004	2	Years	2004-10-20	Recovered	B	E1	Guillain-Barré syndrome	G61.0
28	F	2004	2	Years	2004-11-23	NI	C	EV-C99	Guillain-Barré syndrome	G61.0
29	F	2005	5	Years	2005-03-03	Sequelae	B	E30 - V	Encephalitis or meningoencephalitis	G05.1
30	M	2005	8	Years	2005-02-13	Recovered	B	E30 - V	Guillain-Barré syndrome	G61.0
31	M	2005	7	Months	2005-06-24	Sequelae	B	E30	Guillain-Barré syndrome	G61.0
32	M	2005	1	Year	2005-07-19	Recovered	A	EV-A71 B	Guillain-Barré syndrome	G61.0
33	F	2005	2	Years	2005-07-22	Recovered	A	EV-A71 C1	Encephalitis or meningoencephalitis	G83.3
34	M	2005	1	Year	2005-09-03	NI	A	CVA16	Encephalitis or meningoencephalitis	G04.0
35	M	2005	6	Years	2005-10-10	NI	B	E25	NI	NI
36	M	2005	11	Years	2005-09-19	Recovered	A	CVA3	Unspecified polyneuropathy	G62.9
37	M	2005	4	Months	2005-11-08	Sequelae	B	CVB2	Encephalitis or meningoencephalitis	G05.1
38	M	2005	2	Years	2005-11-08	Recovered	B	CVB2	NI	Não Polio

39	M	2005	1	Year	2005-11-21	Recovered	B	CVB2	Encephalitis or meningoencephalitis	G05.1
40	F	2006	1	Year	2006-02-09	NI	C	EV-C99	Encephalitis or meningoencephalitis	G04.9
41	M	2006	3	Years	2006-02-15	Recovered	B	CVB2	Encephalitis or meningoencephalitis	G04.9
42	M	2006	2	Years	2006-02-26	NI	B	CVB1	NI	NI
43	M	2006	2	Years	2006-10-03	Recovered	A	CVA10	Guillain-Barré syndrome	G61.0
44	M	2006	3	Years	2006-11-02	Recovered	B	E13	Guillain-Barré syndrome	G61.0
45	M	2006	10	Months	2006-12-18	Recovered	A	EV-A71 B	NI	NI
46	F	2006	1	Year	2006-12-15	Recovered	B	CVB5	Guillain-Barré syndrome	G61.0
47	F	2006	6	Months	2006-12-16	NI	B	E18	Myelopathy	G71.2
48	F	2007	2	Years	2007-02-04	Sequelae	B	E11	Unspecified polyneuropathy	G62.9
49	F	2007	1	Year	2007-04-15	Recovered	B	E11	Periodic paralysis	G72.3
50	M	2008	5	Years	2008-04-18	Recovered	B	E11	Other nervous system disorders not classified	A08.3
51	F	2008	2	Years	2008-04-30	NI	A	EV-A71 B	Other nervous system disorders not classified	A08.3
52	M	2008	5	Years	2008-11-14	Recovered	B	E3	Unspecified paralytic syndrome	G83.9
53	M	2009	2	Years	2009-02-08	Recovered	C	EV-C99	Encephalitis or meningoencephalitis	G04.9
54	M	2009	1	Year	2009-03-09	Recovered	B	E11	Unspecified polyneuropathy	G61.9
55	F	2009	3	Years	2009-04-01	Sequelae	B	E11	Unspecified polyneuropathy	G62.9
56	M	2009	10	Years	2009-04-05	Sequelae	C	EV-C99	Guillain-Barré syndrome	G61.0
57	M	2009	4	Years	2009-10-14	Recovered	B	E6	NI	NI
58	F	2009	2	Years	2009-11-11	Recovered	B	E6	Guillain-Barré syndrome	G61.0
59	F	2009	2	Years	2009-11-13	Sequelae	A	CVA5	Unspecified paralytic syndrome	G83.9
60	F	2009	10	Years	2009-12-26	Recovered	B	CVB2	Encephalitis or meningoencephalitis	G04.9
61	F	2010	1	Year	2010-05-02	Sequelae	B	CVB3	Malignant neoplasm of the CNS	C72.9
62	F	2010	9	Years	2010-11-25	Sequelae	B	CVB2	Guillain-Barré syndrome	G61.0
63	M	2011	12	Years	2011-02-21	Recovered	B	E6	Periodic paralysis	G72.3
64	M	2011	1	Year	2011-02-20	Recovered	B	E6	Guillain-Barré syndrome	G61.0
65	M	2011	4	Months	NI	NI	B	E6	NI	NI
66	M	2011	1	Year	2011-08-17	Recovered	B	E11	Other nervous system disorders not classified	G98
67	F	2012	2	Years	2012-04-23	Recovered	A	CVA4	Other nervous system disorders not classified	G98
68	M	2012	13	Years	2012-06-19	Recovered	A	CVA8	Unspecified myoneural disorder	G70.9
69	M	2012	10	Years	2012-09-30	Sequelae	B	CVB5	Guillain-Barré syndrome	G61.0
70	F	2013	2	Years	2013-01-20	Sequelae	-	NTEV	Guillain-Barré syndrome	G61.0
71	F	2013	1	Year	2013-04-23	Recovered	B	E11	Unspecified paralytic syndrome	M46.4
72	F	2013	9	Months	2013-07-02	Sequelae	A	EV-A71 C2	Acute flaccid myelitis	G37.3
73	F	2013	2	Years	2013-09-25	NI	B	CVB3	Guillain-Barré syndrome	G61.0
74	F	2013	1	Year	2013-11-03	NI	A	CVA16	NI	NI
75	M	2013	3	Years	2013-11-08	NI	-	NTEV	NI	NI
76	F	2013	1	Year	2013-12-02	NI	A	CVA16	Other nervous system disorders not classified	G98
77	F	2013	3	Years	2013-12-26	NI	A	CVA16	NI	NI
78	M	2014	1	Year	2014-01-04	Recovered	-	NTEV	Guillain-Barré syndrome	G61.0
79	M	2014	1	Year	2014-01-07	Recovered	C	CVA19	Unspecified intoxication	A05.9

80	M	2014	1	Year	2014-03-03	Recovered	C	EV-C99	Encephalitis or meningoencephalitis	G05.1
81	F	2014	12	Years	2014-02-22	NI	B	E7	NI	NI
82	F	2014	9	Years	2014-07-08	Sequelae	B	E7	Encephalitis or meningoencephalitis	G05.1
83	M	2015	1	Year	NI	NI	B	E11	NI	NI
84	M	2015	1	Year	2015-05-24	Recovered	A	EV-A71 C2	Encephalitis or meningoencephalitis	G04.8
85	F	2015	1	Year	2015-08-07	Recovered	A	CVA16	Periodic paralysis	G72.3
86	F	2015	4	Years	2015-09-02	NI	B	CVB3	NI	NI
87	F	2015	2	Years	2015-09-27	NI	A	CVA4	NI	NI
88	M	2015	1	Year	2015-09-29	NI	C	EV-C99	NI	NI
89	M	2015	2	Years	2015-10-12	Recovered	A	EV-A71 C2	Myelopathy	G99.2
90	M	2016	1	Year	2016-02-11	Recovered	B	CVB3	Guillain-Barré syndrome	G61.0
91	M	2016	2	Years	2016-04-02	NI	B	CVB5	NI	NI
92	M	2016	1	Year	2016-05-16	Recovered	B	E1	Guillain-Barré syndrome	G61.0
93	F	2016	4	Years	2016-07-20	Recovered	B	E16	Unspecified polyneuropathy	G62.9
94	F	2016	2	Years	2016-10-23	NI	B	E30 -V	NI	NI
95	F	2016	1	Year	2016-11-04	Sequelae	B	CVB4	Malignant neoplasm of the CNS	C72.9
96	F	2019	6	Years	2019-01-23	Recovered	B	E6	Encephalitis or meningoencephalitis	G04.9
97	M	2019	5	Years	2019-06-16	NI	C	CVA19	NI	NI
98	M	2019	1	Year	2019-10-01	NI	A	EV-A71	NI	NI
99	M	2019	2	Years	2019-10-20	Recovered	B	CVB3	Periodic paralysis	G72.3
100	M	2021	1	Year	2021-10-25	Recovered	B	E1	Guillain-Barré syndrome	G61.0

ICD: International Classification of Diseases; NTEV: Not typed Enterovirus; NI: No information.
